# Decoupling of sexual signals and their underlying morphology facilitates rapid phenotypic diversification

**DOI:** 10.1002/evl3.302

**Published:** 2022-12-18

**Authors:** James H. Gallagher, David M. Zonana, E. Dale Broder, Brianna K. Herner, Robin M. Tinghitella

**Affiliations:** ^1^ Department of Biological Sciences University of Denver Denver Colorado USA

## Abstract

How novel phenotypes evolve is challenging to imagine because traits are often underlain by numerous integrated phenotypic components, and changes to any one form can disrupt the function of the entire module. Yet novel phenotypes do emerge, and research on adaptive phenotypic evolution suggests that complex traits can diverge while either maintaining existing form–function relationships or through innovations that alter form–function relationships. How these alternate routes contribute to sexual signal evolution is poorly understood, despite the role of sexual signals in generating biodiversity. In Hawaiian populations of the Pacific field cricket, male song attracts both female crickets and a deadly acoustically orienting parasitoid fly. In response to this conflict between natural and sexual selection, male crickets have evolved altered wing morphologies multiple times, resulting in loss and dramatic alteration of sexual signals. More recently, we and others have observed a radical increase in sexual signal variation and the underlying morphological structures that produce song. We conducted the first combined analysis of form (wing morphology), function (emergent signal), and receiver responses to characterize novel variation, test alternative hypotheses about form–function relationships (Form–Function Continuity vs. Form–Function Decoupling), and investigate underlying mechanistic changes and fitness consequences of novel signals. We identified three sound‐producing male morphs (one previously undescribed, named “rattling”) and found that relationships between morphology and signals have been rewired (Form–Function Decoupling), rapidly and repeatedly, through the gain, loss, and alteration of morphological structures, facilitating the production of signals that exist in novel phenotypic space. By integrating across a hierarchy of phenotypes, we uncovered divergent morphs with unique solutions to the challenge of attracting mates while evading fatal parasitism.

**Impact Summary**

How phenotypic novelty evolves is difficult to study because we rarely have the opportunity to observe the earliest stages of diversification. We capitalize on recent diversification of sexual signals in a cricket and show that novel songs have evolved through multiple instances of the restructuring of relationships between songs and the wing morphology that produces them. We characterize multiple newly evolved morphs that produce distinct songs via unique alterations to wings. These novel morphs are effective at attracting mates while avoiding death from a recently introduced parasitoid fly, demonstrating alternate solutions to conflicting selection from mates and natural enemies. Such real‐time work provides a rare opportunity to understand the links between morphology, signal, and fitness following the appearance of novel phenotypes.

The origin of evolutionary novelty is one of the most perplexing yet fundamental processes in the generation of biodiversity. It is difficult to envision how novel traits arise, as many traits are complex and underlain by multiple morphological and physiological components (forms) that interact to dictate trait function (Wagner and Altenberg 1996). Because natural and sexual selection act upon trait function rather than the underlying forms themselves (Arnold 1983; Losos 2011; e.g., selection acts on the bite force exerted by a jaw structure (Alfaro et al. 2005), and the perceived color emitted from a pigmented wing spot; (Grether et al. 2004)), it is necessary to carefully consider the relationships between form and function in order to understand the diversification of complex traits. There are many uses of the term “function” in the study of ecology and evolution, but here we follow Bock (Bock 1980) and use the term to refer to all emergent “physical and chemical properties of a feature arising from its form” (a concept also sometimes referred to as functional “‐consequences” or “‐capabilities”; Losos 2011). Decades of research suggests that phenotypic evolution can be either hindered or facilitated when multiple forms contribute to trait function. On one hand, phenotypic components of complex traits may covary in their expression due to genetic linkage, pleiotropy, and developmental constraints (i.e., phenotypic integration; Cheverud 1996; Cooper et al. 2011; Lande and Arnold 1983; Pigliucci 2003), constraining potential evolutionary trajectories (Klingenberg 2008; Lande and Arnold 1983). But trait complexity may also provide the conditions for novelty to evolve (Navalón et al. 2020). Many‐to‐one mapping (Alfaro et al. 2005; Wainwright et al. 2005) allows multiple phenotypic combinations to reach equivalent functional outcomes (alternate relationships between form and function), and may facilitate the evolution of new paths to fitness peaks (Wainwright 2007).

Evolution that rewires form‐function relationships has long been recognized as facilitating the colonization of new ecological spaces (Heard and Hauser 1995; Mayr 1960; Simpson 1984; Wainwright 2007), but its role in the diversification of sexual signals (i.e., emergent sensory characteristics that receivers experience) is less well understood (Eliason 2018; but see Clark et al. 2011; Eliason et al. 2015; Maia et al. 2013), despite the key role of sexual signal divergence in the generation and maintenance of biodiversity (Gray and Cade 2000; Kopp et al. 2018; Mendelson and Shaw 2002; Niehuis et al. 2013; Panhuis et al. 2001; Pomiankowski and Iwasa 1998; Servedio and Boughman 2017; West‐Eberhard 1983). The diverse, and often conflicting, selective pressures acting on sexual signals (e.g., from intended and unintended receivers; Rosenthal 2017) make them a particularly interesting case in which to study the origins of novelty. Research on the role of sexual selection in signal evolution frequently focuses on the directional and incremental elaboration of ornaments (Bradbury and Vehrencamp 2011; Coyne, Jerry A and Orr, H Allen 2004). However, novel sexual signals (*sensu* Broder et al. 2021a) may also evolve through complex modifications to relationships between form and function, as they often include multiple sensory components (Elias et al. 2005; Hebets and Papaj 2005; Mullen et al. 2007), each of which is produced by underlying morphology (Hebets et al. 2016). It remains unclear whether sexual signal novelty is more often generated through the evolution of exaggerated forms that maintain ancestral form‐function relationships (e.g., Møller 1988), or through morphological innovations that decouple structures from signal properties (Mhatre et al. 2012; Figure 1). However, testing how form‐function relationships are maintained or altered during periods of signal divergence is challenging due to the difficulty of reconstructing the causes and consequences of evolutionary changes that took place long ago and the extreme rarity of opportunities to directly observe signal divergence (Svensson 2019; Svensson and Gosden 2007).

**Figure 1:**
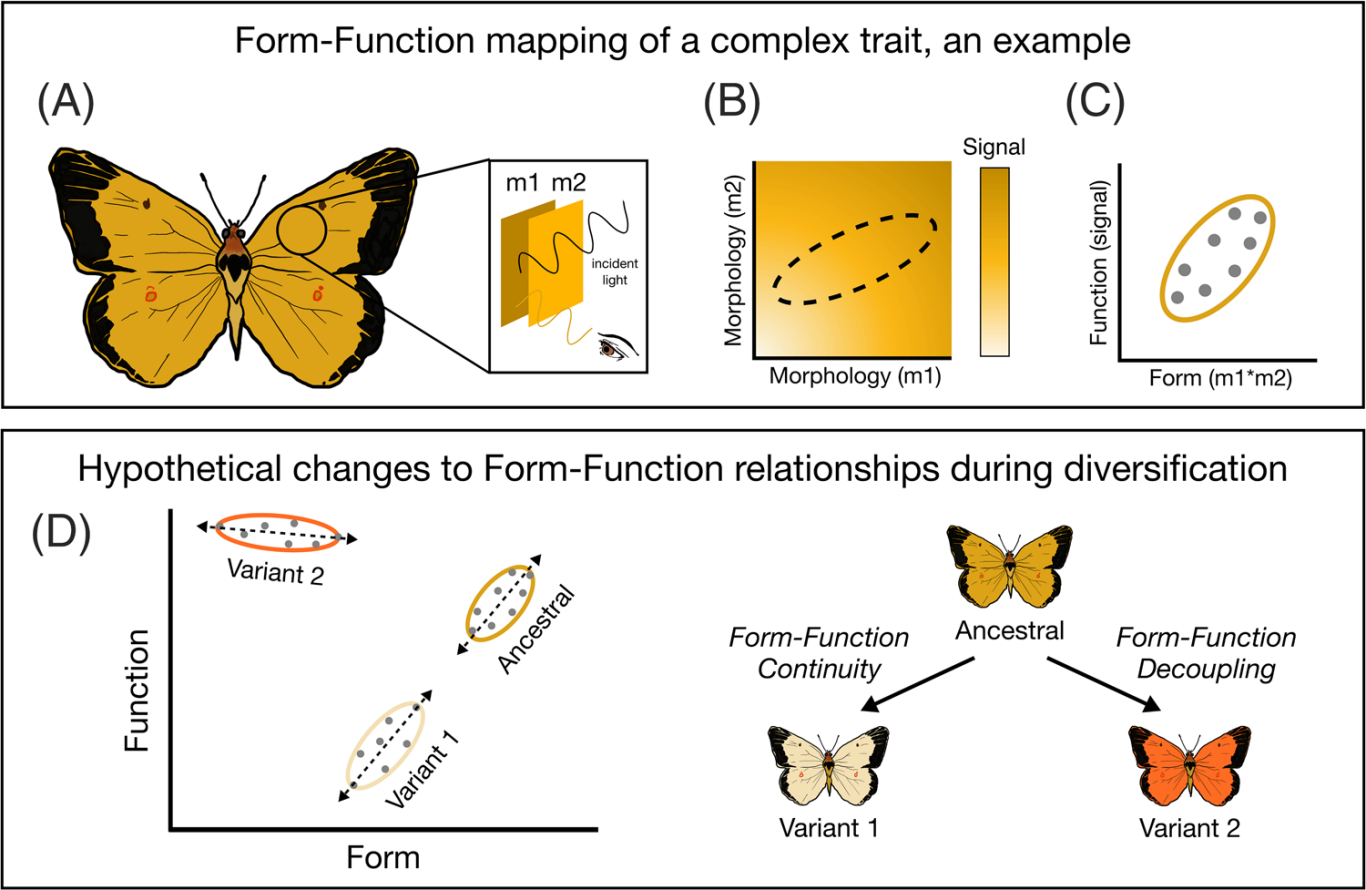
Phenotypic diversification of complex traits depends upon form‐function relationships. (A) A hypothetical scenario where a complex sexual signal (wing coloration; example inspired by Rutowski et al. 2005) is produced by two morphological components: separate cell layers containing different pigments (m1, m2). (B) In the simplest case, two components map independently and additively to dictate function (signal value). There are multiple ways to achieve equivalent signal values within the resulting morphospace. However, functionally related traits are rarely fully independent, but rather are correlated in their expression (indicated by dashed oval), constraining the morphospace into which future phenotypes could evolve. (C) We can similarly visualize form‐function relationships (here, signal‐by‐morphology composite variable). (D) The form‐function plot from C is expanded to include novel variants 1 and 2. Diversification of complex traits can occur while maintaining the established, ancestral form‐function relationships (Form–Function Continuity); variant 1 has the same form‐function relationship (slope) as the ancestral cluster. Alternatively, innovations that rewire form‐function relationships can facilitate diversification by expanding available morphospace (Form‐Function Decoupling); the variant 2 cluster has a novel slope, indicating that the previous form‐function relationship has been changed, allowing the new orange color to evolve.

In this study, we capitalize on the real‐time, rapid evolution of acoustic sexual signals in Hawaiian populations of the Pacific field cricket, which provides a rich opportunity to characterize novel signal variation and test how form–function relationships are reconfigured during bursts of increased signal variation. Male crickets use song in two contexts associated with mating: they produce a long‐distance calling song to attract females from afar and a courtship song to entice females to mount once they are in close proximity (Alexander 1962). These songs convey various information to receivers, with calling song primarily providing species, sex, and location‐based information, and courtship song indicating fitness‐related traits, such as immune function (Tregenza et al. 2006; Zuk et al. 2008; Simmons et al. 2010). Sound is generated when crickets drag the scraper of one wing across the file, a modified wing vein with a row of many continuous small teeth, on the other wing, resonating important veins and structures such as the harp and mirror to create sound (Bennet‐Clark 1999a; Ewing 1989); changes to these structures can affect sound characteristics of the resulting songs (Bennet‐Clark, 1987, 2003; Desutter‐Grandcolas 1998; Koch et al. 1988). However, in Hawaiian populations, male calling songs attract not only potential mates (female crickets) but also a recently introduced parasitoid fly, *Ormia ochracea* (Lehmann 2003). After locating a potential host by eavesdropping on their song, gravid female flies deposit their planidia (specialized larvae) on the male cricket (Adamo et al. 1995). These larvae develop inside the cricket's body cavity and, after devouring the animal's insides, eat their way out in a dramatic scene that harkens childhood nightmares spurred on by the movie “Alien.” In response to this strong selective pressure from the fly (historically 27% of males parasitized; Zuk et al. 1993), separate populations of Hawaiian *T. oceanicus* independently lost sound‐producing structures on their wings, rendering these males obligately silent (named “flatwing” or “silent” males; Pascoal et al. 2014; Tinghitella 2008; Zuk et al. 2006). Silent males are protected from parasitism, but their inability to sing makes mate attraction challenging (Pascoal et al. 2014; Tinghitella 2008; Zuk et al. 2006). Silent crickets do, however, retain ancestral wing movement patterns (stridulation; Rayner et al. 2020) and some vestigial wing structures, features which have been hypothesized to provide an opportunity for the evolution of novel signal function (Bailey et al. 2019). Indeed, in 2017 a new male morph called “purring” was discovered that produces a novel song that attracts mates but evades the parasitoid fly (Tinghitella et al., 2021, 2018). Thus, purring appears to be a novel solution to the conflict between natural and sexual selection in this system; it has since become common across Hawaii (Tinghitella et al. 2021).

While the evolution of two novel morphs in two decades is itself remarkable, the story is far from complete. Since the discovery of purring, we and others have observed a radical increase in sexual signal variation and the underlying morphological structures that produce song (e.g., Rayner et al. 2019). Much of this variation has not been characterized, and the underlying mechanisms and fitness consequences of novel signals remain largely unknown. Here, we conduct the first combined analysis of detailed morphological, song, and fitness data from six Hawaiian populations of *T. oceanicus* to: 1) ask whether male signal diversification supports patterns of evolutionary change through Form‐Function Continuity or Form‐Function Decoupling (Figure 1), 2) characterize groups of males with shared morphology and signals (hereafter, morphs) in order to compare form‐function relationships among morphs, and 3) investigate the fine‐scale morphological mechanisms and fitness trade‐offs underlying novel songs. We find that form‐function relationships between morphology and emergent sexual signals have been rewired, rapidly and repeatedly, through the gain, loss, and alteration of morphological structures, demonstrating how innovations that decouple form and function can facilitate the evolution of novel phenotypes.

## Materials and Methods

### COLLECTION, RECORDING, AND PHOTOGRAPHY

In June 2019, we collected 153 adult males and 172 adult females from six Hawaiian populations: Hilo, Kalaupapa, Manoa, La'ie, Wailua, and Kapa'a (see Table S1 and Supporting Information Methods for sampling details). We housed animals with ad libitum rabbit food, damp cotton (for water), and an egg carton shelter; males were housed individually in 0.5 L plastic deli cups, and females were housed in groups, by site, in 15 L plastic containers. We recorded both calling and courtship songs of individual males using a digital recorder (Marantz PMD620 MKII; Sound United LLC, Carlsbad, CA USA) connected to a RØDE NTG2 Multi‐powered Condenser Shotgun microphone (RØDE Microphones LLC, Long Beach, CA USA) positioned 10 cm above the cricket. For courtship recordings, we added an adult female to the male's container to encourage courtship stridulation. All recordings were conducted indoors during the animals’ natural scotoperiod in rooms lit with only red light. Each recording captured at least one complete bout of uninterrupted song. We took photographs of each male's right wing under natural daylight using a digital SLR camera (Pentax K‐5, Hoya Corp., Tokyo, Japan; Tamron SP 90mm F/2.8 macro lens, Tamron USA Inc., Commack, NY) positioned 10 cm directly above the wing. We gently lifted forewings and pressed them flat on a piece of paper with a printed ruler to facilitate visualization of wing venation. After recording and photographing was complete, we returned all crickets to their collection sites.

### SONG ANALYSIS

We analyzed the second cleanly recorded (without background noise) song from the first bout of continuous song from each male's calling and courtship recordings (see Supporting Information Methods for more detail). We measured nine sound characteristics that capture variation in frequency, amplitude, and broadbandedness (Figure S1). We first determined each song's dominant frequency in Audacity (version 2.3.1, The Audacity Team) using the plot spectrum analysis function (settings: Hanning window, size = 256, log frequency axis). All remaining song analyses were conducted in Logic Pro X (version 10.4.8, Apple Inc., Los Altos, CA USA). We determined the amplitude (RMS level) of each song using Logic Pro X's Level Meter, and then measured the amplitude of six different frequency ranges (Figure S1), chosen because they reflect natural clusters of auditory receptor fibers, and thus “peaks” and “valleys” in *T. oceanicus* hearing ability (Imaizumi and Pollack 1999; Tinghitella et al. 2021). We calculated the relative amplitude of each frequency range by dividing the range's amplitude by the sum of all frequency range amplitudes. We calculated frequency evenness as the additive inverse of the standard deviation of the relative amplitudes of all frequency ranges (Figure S1).

Some song characteristics were correlated with one another, so to understand how songs differed among morphs, we first used principal component analysis (PCA) as a variable reduction technique, collapsing characteristics into fewer axes that describe independent covariance. Because male crickets produce both a calling and courtship song, we conducted separate PCAs on recordings of these distinct signals (Calling Song: N = 143; Courtship Song: N = 112).

### WING MORPHOMETRICS

We chose 14 landmarks (Figure S2, Table S2) based on previous morphometric work in this species (Pascoal et al., 2014, 2017) that capture variation in wing structures known to play a role in sound production (Bennet‐Clark 1999b, 2003; Huber et al. 1989; Prestwich et al. 2000). We placed landmarks on photos of the right wing of each male using tpsDIG2 (v2.3.1; Rohlf 2006; see Supplemental Methods), and used the R package *geomorph* (Adams and Otárola Castillo 2013) to reduce the dimensionality of morphological data using a PCA (gm.prcomp function) that included xy coordinates of all fourteen wing landmarks (N = 131). In addition to the composite morphological variables generated by the PCA, we extracted further information from photographs about specific wing structures by: 1) scoring the presence or absence of the scraper and the mirror, two potentially sound‐altering structures that are sometimes absent in the recently evolved male morphs, 2) measuring the width of the harp (an important resonator in sound production; Bennet‐Clark 1999b, 2003; Prestwich et al. 2000) by calculating the linear distance along the x‐axis between landmarks 5 and 14 (Figure S2), and 3) measuring mirror size by subsetting landmark data to only include points marking the mirror's perimeter (landmarks 6–11, Figure S2), and extracting centroid sizes (gpagen function in *geomorph*).

### MORPHOLOGY AND PERFORMANCE OF NOVEL MORPHS

Because the above morphometric analyses used photographs of live animals (to avoid destructive sampling), we could not examine microstructures in these animals. In 2020 we collected an additional 48 males from the field and removed right wings (22 ancestral (Mo'orea, Hilo), 11 rattling (Hilo), and 15 purring (Manoa)). We used a VHX‐7000 Digital Microscope (Keyence Corporation, Itasca, IL USA) to view and measure the spacing of teeth on the files of purring, rattling, and ancestral males (see Supplemental Methods).

In January 2020, we also collected female *T. oceanicus* and *O. ochracea* (see Tinghitella et al. 2021; Walker 1989) from Hilo for use in behavioral phonotaxis experiments. See Tinghitella et al. 2021 for detailed cricket and fly phonotaxis methods. Briefly, crickets were placed in an arena and played stimuli (purring, rattling, ancestral, and white noise control) in a random order for one minute each or until speaker contact (ancestral always played last). For each phonotaxis trial (*N* = 30 females), we measured whether or not the female cricket exhibited positive phonotaxis and whether they contacted the speaker. Flies (*N* = 8) were tested using the same set of stimuli during their active searching time (dusk) in a 40 × 40 × 61 cm mesh cage where they traveled down (flying and/or walking) towards a speaker broadcasting sound, and we recorded whether they contacted the speaker (yes/no).

To investigate how purring wings produce audible song despite lacking many of the same important sound‐producing structures as silent males, we measured the presence/absence of the scraper on a set of wings from first‐generation, laboratory‐born Wailua males (*N* = 27) that hatched from eggs collected in the field in 2015.

### STATISTICAL ANALYSIS

We performed all statistical analysis using RStudio (RStudio Team 2020, R version 3.6.3; see Supporting Information R script and data). We first visualized form‐function relationships by plotting features of wing morphology against a subset of calling song characteristics using all males in our sample. In order to identify major clusters of variation based on (dis)similarities in both song and wing phenotypes, we subset 59 individuals which had complete morphological and song (both calling and courtship) data (using all individual characteristics for calling and courtship song, plus all wing morphology variables from Table S3), and performed hierarchical clustering using the hclust function (Ward.D2 agglomeration method) in the *factoextra* package (Kassambara et al. 2017). The gap statistic calculated using the hcut and fviz_nbclust functions in *factoextra* identified *k* = 3 as the best‐supported number of phenotypic clusters (morphs). We next used the phenotypic characteristics that defined morphs in the cluster analysis to manually classify a larger sample (*N* = 105) of field‐caught males for which we had both morphological data and recordings of at least one song type (but not necessarily both calling and courtship songs, as was required for inclusion in the initial clustering analysis). To further assess differences among morphs in song and wing variation using this larger data set, we conducted multivariate analysis of variance (MANOVAs) separately for calling song, courtship song, and wing morphology, using the first two composite axes of phenotypic variation (PC1‐2) as response variables and morph as a predictor. We next calculated correlations amongst scaled wing and song traits within‐morphs by calculating Pearson's product moment correlations, and estimated statistical significance using asymptotic t approximations using the rcorr function in the package *Hmisc* (Harrell et al. 2008). A Welch's two‐sample *t*‐test tested for differences in calling song mean dominant frequency between purring males with and without scrapers.

To test for differences in female cricket responses to song variants, we conducted generalized linear mixed models with binomial error structures in *lme4* (Bates et al. 2007) with the presence/absence of phonotactic behavior as the response variables, song variant as a fixed predictor variable, and a female's individual ID as a random effect (to account for individual‐level variation in overall responsiveness). We compared outcomes between song stimuli using pairwise estimated marginal means in the package *emmeans* (Lenth 2021). Due to complete separation in our data when using contact with the playback speaker as a response (no crickets ever contacted a speaker broadcasting white noise), we performed a penalized logistic regression (Firth's bias‐reduced Logistic Regression) in the *logistf* package in R (Heinze et al. 2020).

## Results

### DECOUPLING OF FORM AND FUNCTION DURING PHENOTYPIC DIVERSIFICATION

**Figure 2:**
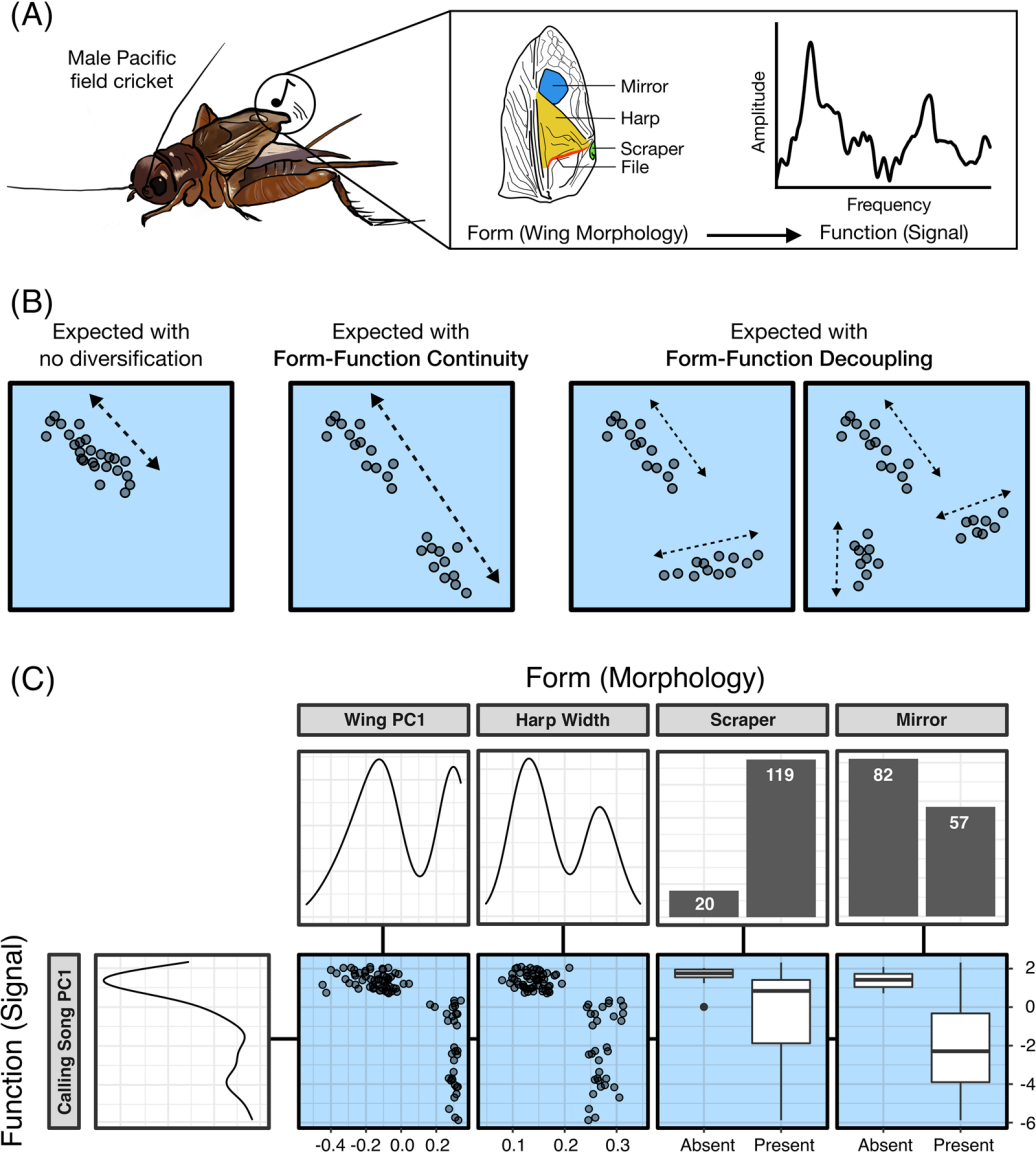
Rapid evolution of sexual signals in *T. oceanicus* provides a rare opportunity to test how complex traits diversify. (A) Rapid evolution of sexual signals in *T. oceanicus* provides a rare opportunity to test how complex traits diversify. Morphological components of wings (mirror, harp, scraper, file) produce mating signals (songs) that vary in spectral characteristics (function, e.g., frequency and amplitude). (B) As described in Figure 1, diversification of sexual signals may occur while maintaining or rewiring form‐function relationships. Hypothetical data display patterns supporting no diversification, diversification with Form‐Function Continuity, and diversification with Form‐Function Decoupling. (C) We investigate form‐function relationships across male Hawaiian *T. oceanicus*, using form to describe wing morphology and function to describe song characteristics (analogous to the use of, for instance, form to describe jaw morphology and function to describe bite force in Alfaro et al. 2005). Calling song recordings and morphometric analyses of field‐caught males showed inconsistent relationships between morphological and signal components across Hawaii (blue boxes), matching patterns shown in panel B that are expected given Form‐Function Decoupling. Form‐function relationships differ among males across Hawaii, as points do not fall along a single axis of covariation. Two important wing structures (scraper, mirror) are present in some sound‐producing males, but absent in others.

To test whether wing morphology and song characteristics covary in similar or different ways among male *T. oceanicus*, we comprehensively measured morphology and signals of male crickets across six populations on four Hawaiian islands. Form‐function relationships were nonlinear across males; a range of wing morphologies produce similar signal values, while highly variable songs result from wings with similar morphology (Figure 2C). These patterns suggest that males produce different sexual signals via alternate pathways between wing and song (Form–Function Decoupling; Figures 1D and 2B). Notably, these nonlinear relationships were evident for all major signal characteristics we investigated (frequency, amplitude, broadbandedness, and a composite of all three, PC1; Figure S1), indicating broadscale decoupling of wing morphology and signal. Having found support for Form‐Function decoupling, we next asked if decoupling occurred once or multiple times, which required us to first identify clusters of males with shared morphology and signals (morphs).

**Figure 3:**
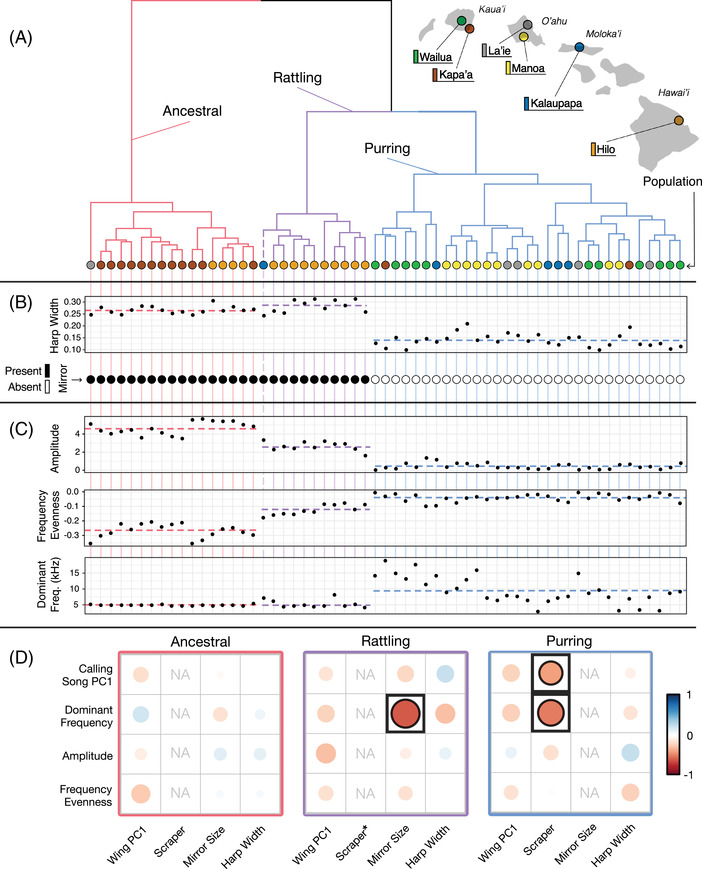
We identified three distinct morphs (ancestral = red, purring = blue, and previously undescribed rattling = purple) with different morphology‐song relationships. (A) Dendrogram of 59 males from across Hawaii, generated via hierarchical clustering based on phenotypic (dis)similarities (Gap statistic: *k* = 3). Leaves of branches are colored by population. Rattling appears unique to the Hilo population; a single individual from Kalaupapa (dashed branch) clustered with rattling, but this was due to uniquely abnormal harp venation, generating songs similar to rattling. (B) Morphology: ancestral and rattling individuals have similar wing morphology, possessing mirrors and wide harps. In contrast, purring males lack mirrors and have reduced harps. (C) Signal: all three morphs differ in amplitude, frequency evenness, and dominant frequency. (D) Differences among morphs in the correlations between wing structures and calling song characteristics illustrate form‐function decoupling (all continuous variables except scraper presence/absence). Bold cells highlight significant morphology‐song relationships, which differ among morphs. The NAs represent cases where within‐morph variation was insufficient for calculating meaningful correlations (e.g., all ancestral males possessed scrapers, while no purring males had mirrors). *Only a single rattling individual was missing a scraper (1/13), so it was impossible to calculate meaningful correlation coefficients between scraper and rattling song characteristics. Note that different morphological features in rattling and purring males (mirror size and scraper presence, respectively) were correlated with the same song component (Dominant Frequency).

We performed hierarchical clustering using 33 measures of song and wing characteristics (Table S2 and Figures S1 and S2) from field‐caught male crickets for which we had complete data (N = 59; calling and courtship song recordings, and wing morphometrics). We uncovered three distinct phenotypic clusters that we define as “ancestral,” “purring,” and a new “rattling” morph that we describe for the first time here (Figure 3A; gap statistic: *k* = 3; see Table S3 for morph‐level means and SDs of all traits). Ancestral males had traits characteristic of *T. oceanicus* from their ancestral range in Australia: wings with fully developed harps and mirrors, and loud, tonal songs with a low dominant frequency (Bennet‐Clark 1999b, 2003). Consistent with previous work (Tinghitella et al. 2018), purring males lacked mirrors altogether, had reduced harps (Figures 3B and S3; similar to silent males; (Zuk et al. 2006)), and produced detectable but dramatically quieter (low amplitude), more broadband songs (high frequency evenness) with variable dominant frequencies (Figures 3C and S3; as in (Tinghitella et al. 2018)). In contrast, the newly discovered rattling morph had categorically different songs from the other two morphs (more power in middle frequencies, intermediate amplitude and frequency evenness; Figures 3C and S3), and differed from ancestral males in song but not wing morphology (as measured by traditional landmarking; Figure 3B). Corroborating the discrete phenotypic groupings revealed by hierarchical clustering (Figure 3), MANOVAs of wing and song variation from a larger sample of field‐caught males (see methods for criteria for inclusion; *N* = 105) showed dramatic differences among morphs (MANOVA, Calling Song: F_4,174_ = 77.8, *p* < 0.0001; Courtship Song: F_4,140_ = 32.2, *p* < 0.0001; Wing Morphology: F_4,204_ = 48.8, *p* < 0.0001; Figure S3). Morph‐level clustering persisted in laboratory‐reared animals after two generations in a common garden, suggesting that rearing conditions have little effect on these distinct phenotypes (Figure S4, Supporting Information Methods; MANOVA; Morph: F_4,248_ = 60.0, *p* < 0.0001; Rearing Treatment: F_2,123_ = 2.0, *p* = 0.14; Morph × Rearing Treatment: F_4,248_ = 0.58, *p* = 0.68).

To examine how form–function relationships differ among the three morphs we just described, we tested how song variation correlates with morphology within each morph. As expected for a trait that has historically been under strong stabilizing selection, we found significantly lower variation in the morphology of ancestral males (Levine's test: F_2,103_ = 21.2, *p* < 0.0001; Figure S5) resulting in weak correlations between morphology and song features (Figure 3D). But both of the derived morphs, purring and rattling, had unique sets of correlations between calling song and morphology components (Pearson's correlations, Table S4; Figure 3D). For instance, dominant frequency varies with mirror size in rattling males but with scraper presence in purring males. This is further evidence that form‐function relationships have been decoupled across Hawaii, as different morphological changes correspond with novel variation in the same song characteristic.

Collectively, we see strong evidence for Form–Function Decoupling in this system (Figures 2 and 3). Novel broadband, attenuated songs are produced by two separate wing types (purring and rattling), and it appears that males with similar wing morphology can produce dramatically different songs (rattling and ancestral; Figure 3A–C). These findings raise additional questions about the mechanistic basis of morphology‐signal novelty that has evolved over the last two decades. Mirror size does appear to influence the frequency of rattling calls to some degree (Figure 3D), yet there is much overlap in wing morphology (including mirror size) of ancestral and rattling males despite their categorically distinct songs (Figure S3C). This suggests mirror size cannot explain the dramatic differences between rattling and ancestral songs (Figure S4B). How can morphs that appear to overlap in wing morphology (ancestral and rattling) produce non‐overlapping signals (Figure S3C)? How do purring wings produce audible song despite lacking many of the same important sound‐producing structures as silent males (Tinghitella et al. 2018; Zuk et al. 2006)?

### MORPHOLOGY AND PERFORMANCE OF NOVEL MORPHS

**Figure 4:**
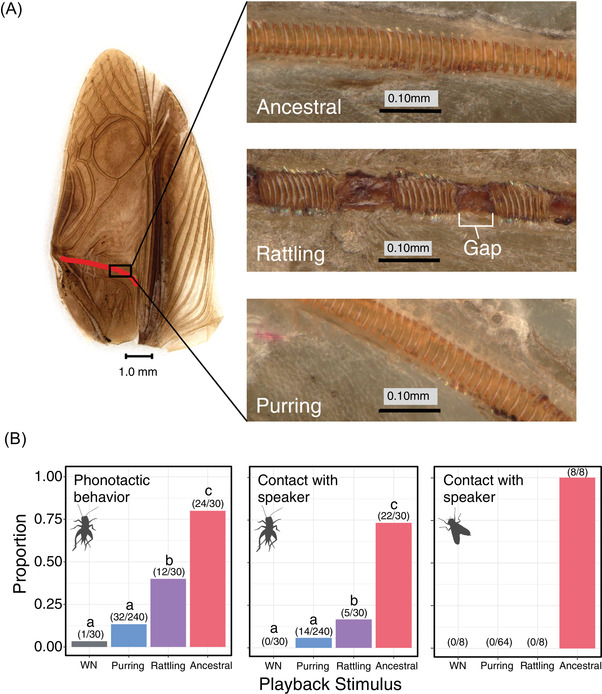
Rattling song is produced via discrete modification of an existing structure (the file), and provides a solution to the problem of attracting mates while avoiding parasitism. (A) Unlike ancestral (N = 0/22) and purring males (N = 0/15), all rattling males (N = 11/11) had distinct gaps between groups of teeth on the file (red line on example rattling wing; see Figure S3 for ancestral and purring example wings). (B) In Hilo (where rattling exists but purring does not), rattling was more attractive to female crickets than purring and white noise (WN), but less attractive than ancestral calling song (attractiveness measured as phonotactic behavior and contact with speaker). Rattling was unattractive to parasitoid flies—they did not contact the speaker when rattling song was played, but did when ancestral song was played.

To further understand the morphological mechanisms producing novel signals, and because our morphometrics above did not explain the discrete differences between rattling and ancestral songs, we used digital microscopy to compare microstructures on the underside of the wing that are not measured by common landmarking approaches (Pascoal et al., 2014, 2017). Crickets make sound by moving the scraper of one wing across the file (a modified vein containing continuous microscopic teeth) on the other wing (Bennet‐Clark 1999b; Ewing 1989). All rattling wings, but no purring or ancestral wings, had distinct gaps where file tooth development was disrupted (Figure 4A). Spacing among individual teeth contributes to song differences among cricket species (Desutter‐Grandcolas 1998; Montealegre‐Z 2009; Montealegre‐Z et al. 2011) and is typically invariant within species due to stabilizing selection from choosy females (Duncan et al. 2021). However, the pattern of larger gaps between groups of teeth seen here in rattling males has not been documented in crickets before, to our knowledge. Importantly, gaps in the teeth of the file were immediately apparent upon eclosion to the adult stage in lab‐reared rattling males, and the proportions of rattling males were remarkably consistent when comparing field‐sampled (N = 8/31 males; 26% rattling) and lab‐born animals (N = 13/48; 27% rattling), demonstrating that gaps are not likely caused by environmental differences or age‐related wear. Detailed song analysis revealed categorically different courtship songs between groups of lab‐born males that differed only in the presence of file tooth gaps, further implicating tooth gaps in the generation of the distinct rattling song (Figure S6; *t*‐test: *t* = 6.68, *df* = 7.88, *p* = 0.0002, *n* = 10). It is possible that the wing movements of novel male morphs like rattling differ from that of ancestral males and that this could contribute to song differences. Note, however, that both purring and silent males retain the stridulatory patterning of ancestral males (Rayner et al. 2020; Tinghitella et al. 2018). Our discovery of gaps in the file likely explains why rattling males produce dramatically different songs from ancestral males, despite largely overlapping wing morphology (Figures 3B and S3).

We next tested whether rattling song functions as a signal within a mating context, or as a cue to eavesdropping parasitoids, by measuring responses of female crickets and flies to playback stimuli (ancestral, rattling, purring, and white noise, following Tinghitella et al. 2021) in the population where rattling exists (Hilo). We found that female crickets, but not flies, are attracted to rattling songs (Figure 4B; Table S5), suggesting that rattling is a private mode of communication (with regard to the primary eavesdropper, *O. ochracea*), as has recently been shown for purring (Tinghitella et al., 2021, 2018). Because purring and rattling appear to be two alternative solutions to shared, conflicting natural and sexual selection pressures, selection may increase the frequencies of these morphs in the populations where they are found.

**Figure 5:**
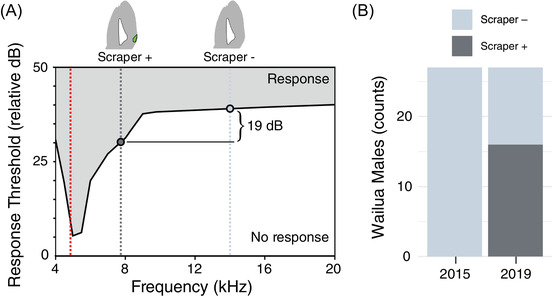
Purring males with scrapers produce lower frequency (closer to ancestral), and therefore more detectable, songs; this structure is rapidly increasing in prevalence in a population where it was previously absent. (A) In this female response threshold figure (adapted from Hoy et al. 1982), the shaded gray section shows the signal space that elicits female response. Because the amplitudes required to trigger female responses vary across frequencies, changes to mean frequency affect detectability. Dotted lines show the median calling song dominant frequency for purring males with and without scrapers (gray vertical lines), and for ancestral males (red vertical line). Purring males without scrapers would need to produce louder songs than those with scrapers to elicit female response. (B) The proportion of Wailua males with scrapers increased from 0% (0/27) to ∼60% (16/27) between 2015 and 2019.

We then turned to the morphology that underlies the production of purring songs. The morphology of purring wings is very similar to that of silent male wings (Tinghitella et al. 2018). In our above analyses (Figure 3D), the scraper was the only wing feature significantly correlated with purring calling song variation and was specifically associated with overall variation (PC1) and dominant frequency (Figure 3D). Further analysis of frequency differences among purring males revealed that individuals with scrapers had calling songs with dramatically lower median dominant frequencies than scraperless males (scraper present: 7.6 kHz, scraper absent: 13.6 kHz; *t* = −4.66, *df* = 13.6, *p* = 0.0004; Figure 5A). Crickets are more sensitive to certain sound frequencies than others (Hoy et al. 1982), so frequency properties of a signal will affect its perceived loudness to the animal. Therefore, a shift in frequency, even without a change in overall amplitude, impacts the ability of a stimulus to elicit a behavioral response from females. The lower dominant frequencies of purring male songs with scrapers fall in a range to which female crickets are more sensitive (closer to ancestral song frequencies; Bennet‐Clark 2003). Based on previously published behavioral response thresholds (Hoy et al. 1982), tones with dominant frequencies matching those of scraperless purring males would need to be approximately 19 dB louder to elicit a positive female response than those of males with scrapers (Figure 5A, adapted from Hoy et al. 1982; note that perceived differences by female crickets may be less extreme because purring songs are not pure tones, unlike stimuli used to generate response curves). Given the low overall amplitude of purring songs (many within 5 dB of background noise in the field), even subtle differences in detectability by females could determine which males’ displays can operate as signals and which cannot (functionally silent). Identifying where exactly this sensory threshold lies will require additional neurophysiological and behavioral studies of receivers.

Interestingly, the scraper has been implicated as a potential difference between silent and purring morphs (Tinghitella et al. 2018), which have historically been classified based on morphology (lack of many wing structures) and/or detectability by human observers. The effects of song frequency changes on perceived loudness that we describe above would not only influence intended female recipients, but also human researchers (Gelfand 2001; Jackson et al. 1999). Therefore, having identified the substantial effect of scraper on calling song characteristics, we assessed whether the increased abundance of purring males recently observed in some populations (Tinghitella et al. 2021) has coincided with an increase in scraper presence. We measured the presence or absence of scrapers on archived male wings from a population that was historically silent (10 years ago; Zuk et al. 2018) but was predominantly purring in our 2019 sample. In 2015, no sampled males (0/27) had scrapers, while ∼60% (16/27) had scrapers in 2019 (Figure 5B). This reappearance of scrapers in Wailua—whether due to mutation, gene flow, or standing genetic variation—suggests that over only a 4‐year period (∼16 generations), the sound produced during male displays may have evolved to become more detectable, potentially restoring sexual signal function (purring; Tinghitella et al. 2018).

## Discussion

**Figure 6:**
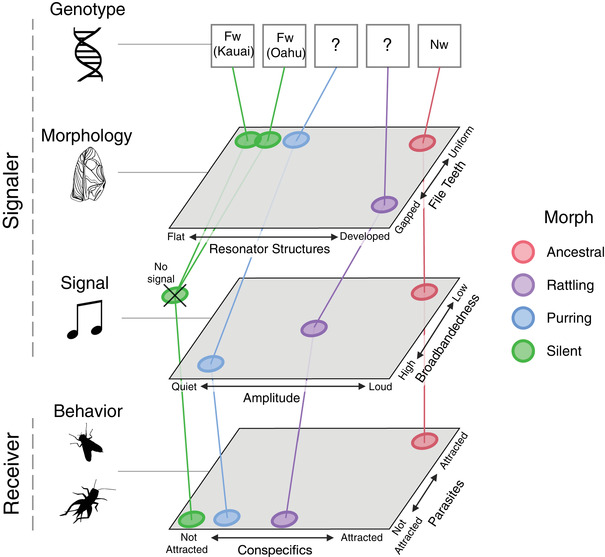
A schematic of the causes and consequences of sexual signal diversity in Hawaiian *T. oceanicus* showing how signals emerge through the interacting levels of genes, morphology, signal, and behavior (inspired by Figure 1 in Eliason 2018), necessitating an integrative research framework. We synthesize our findings from this paper and others (Bennet‐Clark 2003; Pascoal et al. 2014; Tinghitella 2008; Tinghitella et al., 2021, 2018; Zuk et al. 2006) to place four prominent male morphs relative to each other on these levels (we simplify to two, out of many possible dimensions). Clearly, signals are generated through alternate paths across morphs, as indicated by non‐parallel connections between levels. Genotype abbreviations: “Fw” = flatwing, “Nw” = normal wing, “?” = unknown.

By integrating data across a hierarchy of phenotypes and resulting performance, we show that changes to multiple different wing structures have resulted in the evolution of novel acoustic signals (purring and rattling), each of which appears effective at attracting mates while avoiding fatal parasitism (Tinghitella et al. 2021). Divergent male morphs of the rapidly evolving Hawaiian populations of *T. oceanicus* achieve fitness through alternate relationships between morphology and signal, illustrating how the process of Form‐Function Decoupling (Figure 1) may be important during the evolution of novel sexual signals, as is well documented for ecological traits (Heard and Hauser 1995; Mayr 1960; Simpson 1984; Wainwright 2007). The causes and consequences of evolution involving complex restructuring of relationships among phenotypic components may be mischaracterized by studies that do not jointly analyze form and function. We bridge previous work in this system on the functional genetics of wing morphology (Pascoal et al., 2014, 2020; Tinghitella 2008; Zhang et al. 2021) and the fitness consequences of signal variation (Tinghitella et al., 2021, 2018; Zuk et al. 2006), and we illustrate this in Figure 6. In the same way that independent mutations converged upon wings lacking sound‐producing structures (silent; Pascoal et al. 2014), we show alternate routes from morphology to novel signals (non‐parallel connections between Morphology and Signal in Figure 6). Many‐to‐one mapping is inherent in complex traits, allowing multiple routes from form to function (Wainwright et al. 2005); it may facilitate phenotypic innovation and expand the number of possible evolutionary trajectories (Thompson et al. 2017). Therefore, in this system, future directional selection for song characteristics that differ from ancestral song (and protect against fatal parasitism) may result in further morphological divergence among morphs (Lande 1980; Thompson et al. 2017) due to the fact that they produce signal variation through alternate morphological pathways. Finally, selection from receivers (Behavior level, Figure 6) is critically important in the diversification of sexual signals (Page et al. 2014; Rosenthal 2017;Broder et al. 2021a; Rosenthal and Ryan 2022), and strong natural selection against an ancestral signal (Tinghitella et al. 2021) coupled with relaxed sexual selection (Bailey and Zuk 2008; Tinghitella and Zuk 2009), as we see in Hawaiian *T. oceanicus*, may facilitate signal novelty.

The types of mechanistic changes that could theoretically result in form‐function decoupling are finite; forms could either be gained, lost, or altered in ways that break ancestral relationships amongst phenotypic components, resulting in novelty (Broder et al. 2021a; Starrett et al. 2022). The well‐documented evolution of silent *T. oceanicus* occurred through a major mutation that resulted in the loss of important resonator structures on male wings (mirror, scraper; (Pascoal et al. 2014; Tinghitella 2008). Bailey et al. (2019) demonstrated that signal loss in silent crickets has led to increased variation in vestigial wing structures, and proposed that this release of variation could facilitate novel signal values if and when song is restored. Males from the population of Wailua, where silent crickets were first discovered, are now producing novel, attenuated songs (purring; Figure 3), suggesting that Bailey's prediction may be coming to fruition. While we do not know the genetic architecture underlying purring, our data implicate the gain of the scraper, a previously lost structure, as a potential mechanism for signal restoration in this population (Figure 5). Additionally, our results on the morphological underpinnings of the newly discovered rattling morph (Figure 4A) points to a categorical alteration of an existing wing structure (the file) in the generation of a novel signal. Over very short timescales, phenotypic evolution in Hawaiian *T. oceanicus* appears to provide examples of gain, loss, and alteration of forms modifying signal function, however additional work is needed to fully resolve how these morphs relate to one another.

We found support for Form‐Function Decoupling (Figures 1 and 2) here, but sexual signal novelty can also evolve via Form‐Function Continuity, as is likely in the cases of the diversification of the avian syrinx (Kingsley et al. 2018) and song in *Gryllus* field crickets (Caetano and Beaulieu 2020). Indeed, there may also be a case of signal novelty evolving via Continuity in *T. oceanicus*. The wing of the recently discovered “small‐wing” morph (Rayner et al. 2019) appears to produce a new song but retain all of the structures on the ancestral wing (scaled down to a smaller size), though relationships between wings and songs of this morph should be analyzed in further detail. It would be interesting to explore the relative importance of Form‐Function Continuity and Form‐Function Decoupling in the evolution of sexual signal novelty using multi‐species comparative studies.

Understanding the evolutionary processes that facilitate rapid phenotypic diversification may provide insight into the earliest stages of animal signal radiations, which remains somewhat of a black box in evolutionary biology despite much emphasis on the importance of sexual signal radiations in generating diversity (Boake 2005; Coyne, Jerry A and Orr, H Allen 2004; Mendelson and Shaw 2005; Seehausen et al. 1997; Wilkins et al. 2013). Changes to the many selective pressures acting upon a given trait can strongly influence the ability for variation to be generated and persist within populations. One well‐documented change in selective pressures is weakened selection on a previously optimal phenotype (relaxed selection; Lahti et al. 2009). Relaxed selection allows for the accumulation of genetic and phenotypic variation, which may provide the material that other selective pressures can act on (Lahti et al. 2009). Sexual selection appears to be weakened in Hawaiian *T. oceanicus*, as females there are more likely to mount non‐ancestral males than are females from the crickets’ native range in Australia (Tinghitella and Zuk 2009), a phenomenon that is well‐documented in small populations where the initial costs of being choosy following colonization are heightened (Kaneshiro 1980; McPhail 1969; Shaw and Lugo 2001; Tinghitella and Zuk 2009). Indeed, the novel morphs that we describe here should have greater success if females are willing to accept a broad range of signal values (Figure 4B; Tinghitella et al. 2021). At this early stage of diversification, we find that females do not systematically prefer particular purring songs with specific acoustic properties (Tinghitella et al. 2021). While the sensory capabilities of *T. oceanicus* from Australia have been studied (Hoy et al. 1982), it's possible that these capabilities have changed in Hawaii, broadening the range of acceptable signal values. Alternatively, selection pressures may become relaxed if the information content of a signal changes or becomes less relevant to receivers. Beyond their efficacy in the important task of mate location, we know little about if and how the information content of these novel signals differs from that of the ancestral songs. Future studies should test relationships between signal variation and male quality in these morphs.

Selective pressures may also be reversed, where a previously advantageous phenotype becomes strongly selected against (reversed selection; Rayner et al. 2022). In Hawaiian populations of *T. oceanicus*, the arrival and proliferation of the fly changed the selective landscape so that net selection on ancestral song was reversed; selection from flies against males producing ancestral song may have allowed for multiple successful new morphs to become quickly established, as nearly any deviation from the previously optimal ancestral signal may increase male fitness (Figure 4B; Tinghitella et al. 2021). Relaxed or reversed selection may be a broadly important precursor for the generation and success of novel variation in complex traits.

The novelty we discovered points to ongoing phenotypic diversification across Hawaii. Because we found significant differences in performance among signal variants (Figure 4B; Tinghitella et al. 2021), and gene flow is ongoing among islands (Zhang et al. 2021), we can now watch evolution in action. Real‐time research on rapid evolution, as we present here, provides unique opportunities to test the immediate fitness consequences of novel forms within the very environments in which they first appear. Close observation of emerging phenotypic variation in Hawaiian *T. oceanicus* allows for a deeper understanding of which phenotypic innovations are successful, which are evolutionary dead ends (insights missed by retrospective approaches; Rabosky 2017), and whether novel phenotypes arise that rewire form‐function relationships in even more successful and surprising ways.

## AUTHOR CONTRIBUTIONS

J.G., D.Z., and R.T. conceived of and designed the study. J.G., D.Z., E.D.B., and R.T. wrote the manuscript. R.T., J.G., and D.Z. funded the study. J.G. and D.Z. performed analyses and visualizations. J.G., D.Z., E.D.B., B.H., and R.T. contributed to data collection and manuscript editing.

## Supporting information


**Figure S1**. Annotated power spectrum of a single sample calling song displaying the nine sound characteristics measured in each calling and courtship song. “Dominant frequency” is the frequency with the greatest acoustic power. “Amplitude” is a measure of how loud the song is across all frequencies (using RMS level). Songs were spectrally divided into six frequency ranges (A‐F), chosen because they represent natural clusters of auditory receptor fibers in *T. oceanicus*, indicating hearing sensitivity at different frequencies (Imaizumi and Pollack 1999). We divided the amplitude of each frequency range by the sum of all ranges’ amplitudes to determine the proportion of acoustic energy (“relative amplitude”) in each frequency range. We took the standard deviation of all relative amplitude ranges (A‐F), multiplied by −1, as a measure of how evenly distributed the acoustic energy is across the song's frequency spectrum (a measure of how broadband the sound is). We called this final characteristic “frequency evenness” (Formula: ‐(relative amplitude of ranges A,B,C,D,E,F)).
**Figure S2**. Example wing with landmarks placed at their respective locations. See Table S1 for location descriptions. Landmark locations adapted and modified from (Pascoal et al. 2014, 2017).
**Figure S3**. Example wings (A) and courtship songs (B) of ancestral, rattling, and purring males. Structures highlighted are important in sound production, and thus changes to them may alter (or even prevent) song (Desutter‐Grandcolas 1998; Bennet‐Clark 1999, 2003; Zuk et al. 2006; Montealegre‐Z et al. 2009, 2011; Tinghitella et al. 2018; Duncan et al. 2021). Ancestral and rattling males both have fully intact harps, mirrors, and scrapers, while purring males have reduced harps and no mirrors. Rattling males have unique gaps between groups of teeth on the file (see Figure 4A). C) Principal component analyses for calling songs, courtship songs, and wing morphology of ancestral, rattling, and purring phenotypes using a sample of field‐caught males manually classified to morph using diagnostic phenotypic characteristics from the cluster analysis (N=105). Songs differ among morphs for both calling (MANOVA: F_4,174_=77.8, p<0.0001) and courtship songs (MANOVA: F_4,140_=32.2, p<0.0001), as well as morphology (Wing Morphology: F_4,204_=48.8, p<0.0001). Ellipses represent 90% confidence intervals. PC1 largely captures the extent to which a song is ancestral‐like. Song characteristics of rattling are in many ways intermediate to those of ancestral and purring songs.
**Figure S4**. Morph‐level differences in wing morphology (based on geometric morphometrics of 14 landmarks of the dorsal side of the wing; Figure S2, Table S2) were robust to differences in rearing environment. Morph was strongly predictive of morphological differences (MANOVA; Morph: F_4,248_=60.0, p<0.0001) while rearing treatment and the interaction between morph and rearing treatment were not (MANOVA: Rearing Treatment: F_2,123_=2.0, p=0.14; Morph x Rearing Treatment: F_4,248_=0.58, p=0.68). The consistency of morphological differences across morphs within a common‐garden, lab context suggests that differences have a genetic basis.
**Figure S5**: Differences in morphology among morphs. A) Standard deviations of wing morphometric variables show greater levels of morphological variation amongst alternate morphs (purring and rattling) compared to wings of ancestral males (Levine's test: F_2,103_=21.2, p<0.0001). B) Though there is a statistically significant difference in mirror size between rattling and ancestral wings, it cannot explain the dramatic differences in song between morphs. There is much overlap in mirror size between morphs, and morph‐level differences became non‐significant when two outlier rattling males (with small mirrors) were removed from the dataset. Additionally, a larger resonator (mirror) should be associated with lower frequency sound (Bennet‐Clark 1999), but we found the opposite pattern in rattling, but not ancestral, males—rattling songs have higher mean frequency than ancestral songs.
**Figure S6**. Courtship songs of second‐generation, lab‐reared males differed greatly depending on the presence of file tooth gaps, a diagnostic characteristic of the rattling morph. Males with tooth gaps (rattling males) had significantly greater courtship song PC1 values than males without gaps (ancestral males; T‐test: t=6.68, df=7.88, p=0.0002, n=10). Courtship songs and wings were analyzed within two weeks of males eclosing to the adult stage in a common‐garden, lab setting, removing most environmental/age effects on phenotypic differences.
**Table S1**. Sampling sites across Hawaii used in this study.
**Table S2**. Descriptions and notes for wing landmarks.
**Table S3**. Means and standard deviations of trait values of ‘ancestral’, ‘rattling’, and ‘purring ’individuals from the dataset used in Figure S3.
**Table S4**: Correlations (r) between song and wing traits within A) ancestral (N = 23), B) rattling (N = 13), and C) purring males (N = 69) from the dataset used in Figure S3. P‐values shown in parentheses. All variables are continuous except scraper (presence/absence). Bold cells highlight significant morphology‐song relationships. Note that we show correlations for scraper presence in rattling males, but these patterns are driven by a single rattling male that lacked a scraper and should be interpreted with caution.
**Table S5**. Pairwise comparisons of the effects of song stimuli (purring, rattling, ancestral, and white noise (WN)) on A) female cricket phonotactic behavior and B) contact with playback speaker. Comparisons made with estimated marginal means, and contrasts from Firth's Penalized Logistic Regression for phonotactic behavior and contact with speaker models, respectively (N = 30 females from Hilo).

## Data Availability

All data and R scripts are publicly available on Dryad: https://doi.org/10.5061/dryad.sf7m0cg9j.
