# Supplementary material for: Decoupling of sexual signals and their underlying morphology facilitates rapid phenotypic diversification
Source: Evol Lett. 2022 Dec 18;6(6):474–89. doi: 10.1002/evl3.302 (PMC9783451; doi:10.1002/evl3.302)
Supplement: Supplementary file 1 — Figure S1. Annotated power spectrum of a single sample calling song displaying the nine sound characteristics measured in each calling and courtship song. “Dominant frequency” is the frequency with the greatest acoustic power. “Amplitude” is a measure of how loud the song is across all frequencies (using RMS level). Songs were spectrally divided into six frequency ranges (A‐F), chosen because they represent natural clusters of auditory receptor fibers in T. oceanicus, indicating hearing sensitivity at different frequencies (Imaizumi and Pollack 1999). We divided the amplitude of each frequency range by the sum of all ranges’ amplitudes to determine the proportion of acoustic energy (“relative amplitude”) in each frequency range. We took the standard deviation of all relative amplitude ranges (A‐F), multiplied by −1, as a measure of how evenly distributed the acoustic energy is across the song's frequency spectrum (a measure of how broadband the sound is). We called this final characteristic “frequency evenness” (Formula: ‐(relative amplitude of ranges A,B,C,D,E,F)). Figure S2. Example wing with landmarks placed at their respective locations. See Table S1 for location descriptions. Landmark locations adapted and modified from (Pascoal et al. 2014, 2017). Figure S3. Example wings (A) and courtship songs (B) of ancestral, rattling, and purring males. Structures highlighted are important in sound production, and thus changes to them may alter (or even prevent) song (Desutter‐Grandcolas 1998; Bennet‐Clark 1999, 2003; Zuk et al. 2006; Montealegre‐Z et al. 2009, 2011; Tinghitella et al. 2018; Duncan et al. 2021). Ancestral and rattling males both have fully intact harps, mirrors, and scrapers, while purring males have reduced harps and no mirrors. Rattling males have unique gaps between groups of teeth on the file (see Figure 4A). C) Principal component analyses for calling songs, courtship songs, and wing morphology of ancestral, rattling, and purring phenotypes using a s [file EVL3-6-474-s001.docx]

Supplementary Material


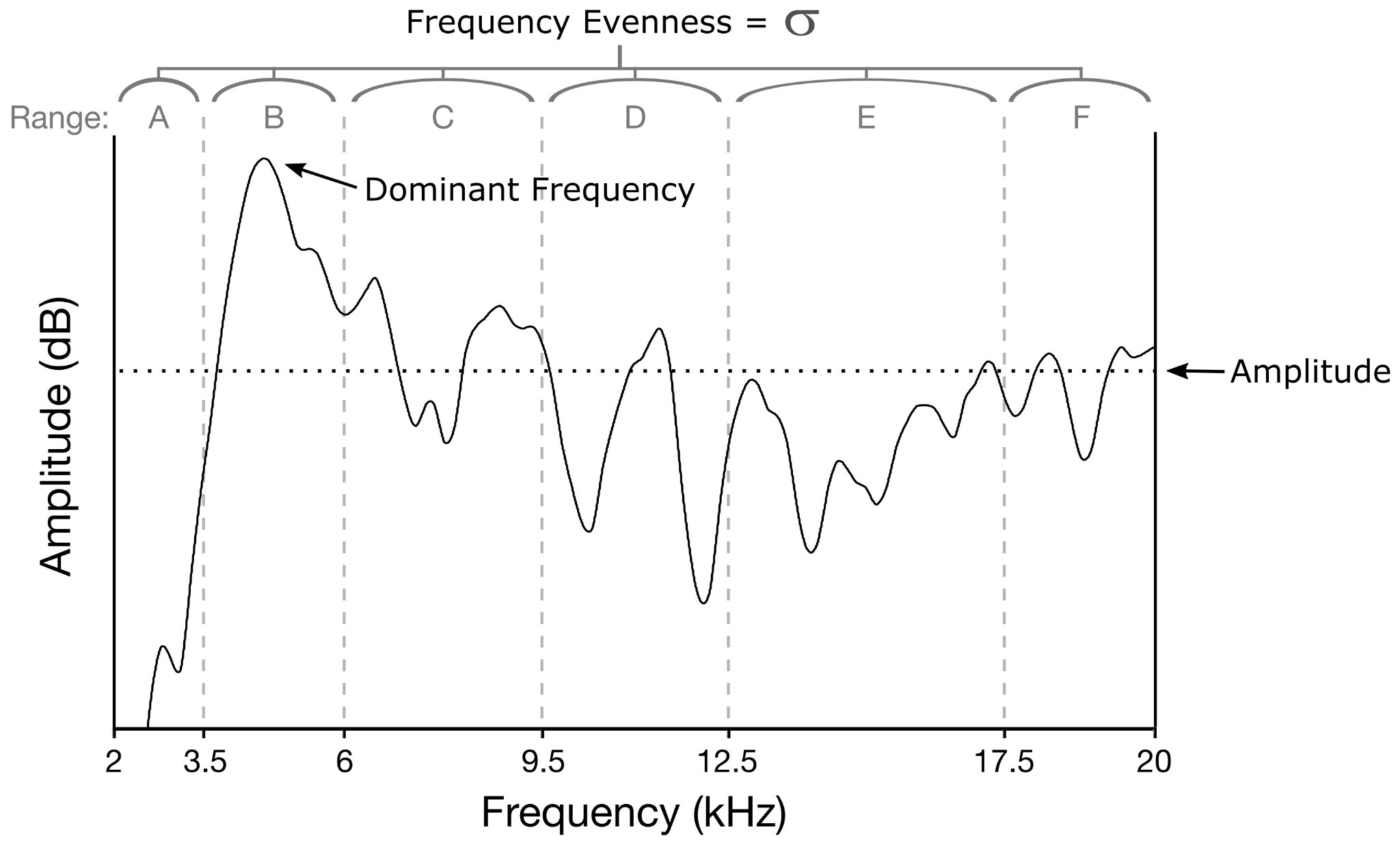


Figure S1. Annotated power spectrum of a single sample calling song displaying the nine sound characteristics measured in each calling and courtship song. “Dominant frequency” is the frequency with the greatest acoustic power. “Amplitude” is a measure of how loud the song is across all frequencies (using RMS level). Songs were spectrally divided into six frequency ranges (A-F), chosen because they represent natural clusters of auditory receptor fibers in *T. oceanicus*, indicating hearing sensitivity at different frequencies [(Imaizumi and Pollack 1999)](https://paperpile.com/c/7lU1tu/KhMyV). We divided the amplitude of each frequency range by the sum of all ranges’ amplitudes to determine the proportion of acoustic energy (“relative amplitude”) in each frequency range. We took the standard deviation of all relative amplitude ranges (A-F), multiplied by -1, as a measure of how evenly distributed the acoustic energy is across the song’s frequency spectrum (a measure of how broadband the sound is). We called this final characteristic “frequency evenness” (Formula: -(relative amplitude of ranges A,B,C,D,E,F)).


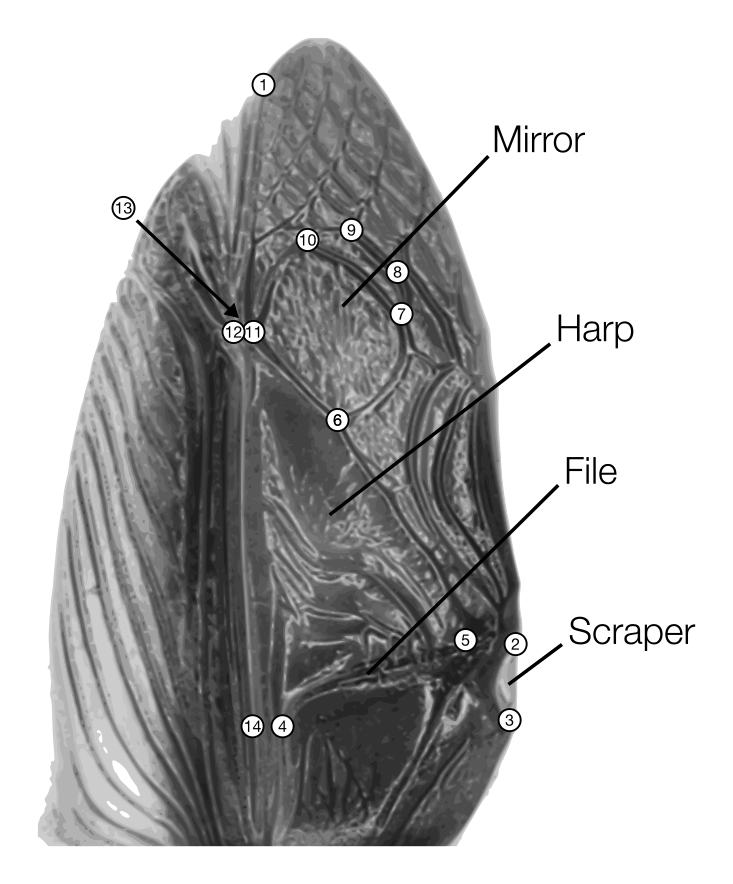


Figure S2. Example wing with landmarks placed at their respective locations. See Table S1 for location descriptions. Landmark locations adapted and modified from [(Pascoal et al. 2014, 2017)](https://paperpile.com/c/7lU1tu/opxk+yN9fo).


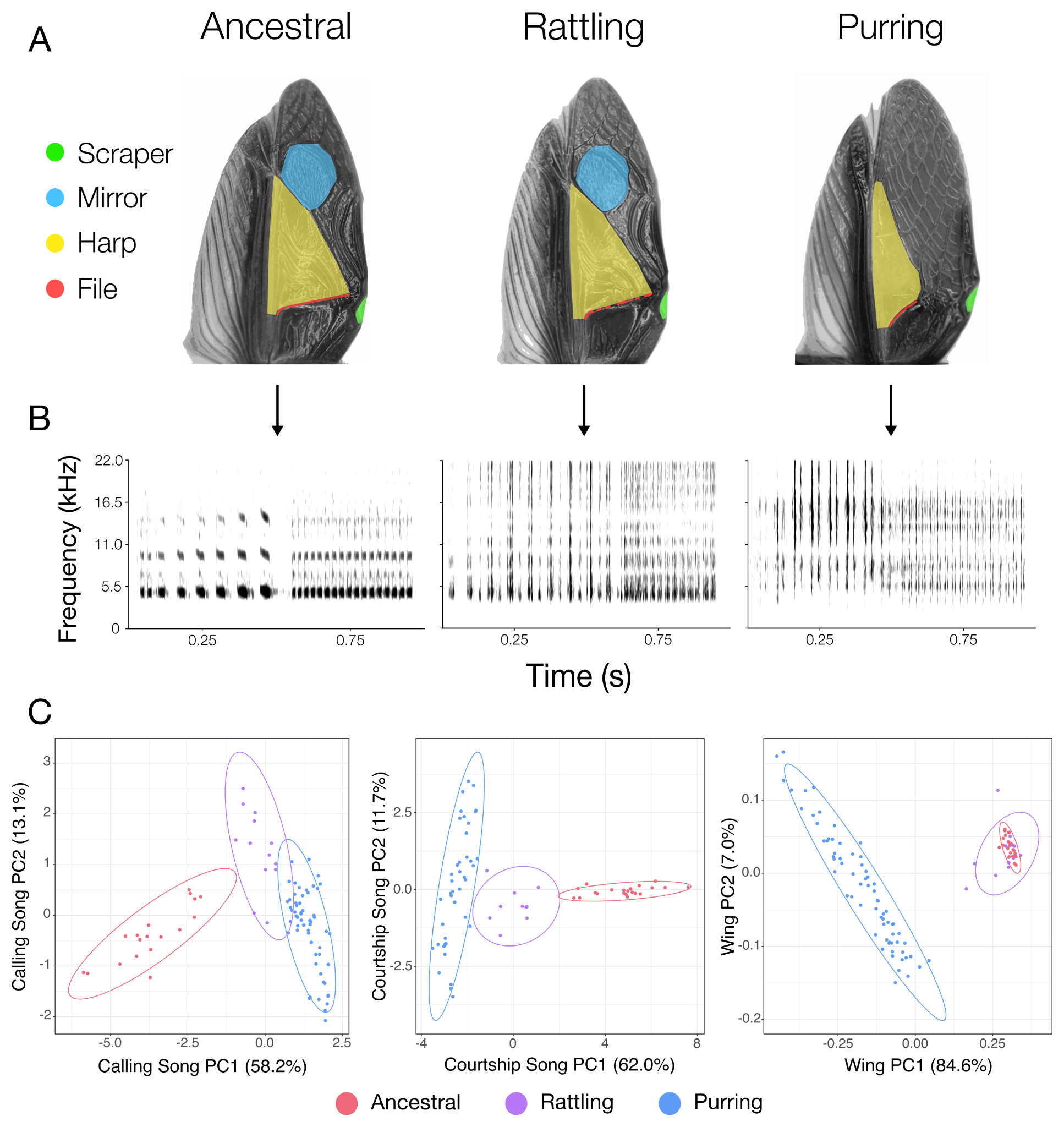


Figure S3. Example wings (A) and courtship songs (B) of ancestral, rattling, and purring males. Structures highlighted are important in sound production, and thus changes to them may alter (or even prevent) song [(Desutter-Grandcolas 1998; Bennet-Clark 1999, 2003; Zuk et al. 2006; Montealegre-Z et al. 2009, 2011; Tinghitella et al. 2018; Duncan et al. 2021)](https://paperpile.com/c/7lU1tu/6bYqq+HQIbB+fwgaC+6YLRP+OVOuV+Y36g3+oMWf+PeYAZ). Ancestral and rattling males both have fully intact harps, mirrors, and scrapers, while purring males have reduced harps and no mirrors. Rattling males have unique gaps between groups of teeth on the file (see Figure 4A). C) Principal component analyses for calling songs, courtship songs, and wing morphology of ancestral, rattling, and purring phenotypes using a sample of field-caught males manually classified to morph using diagnostic phenotypic characteristics from the cluster analysis (N=105). Songs differ among morphs for both calling (MANOVA: F_4,174_=77.8, p<0.0001) and courtship songs (MANOVA: F_4,140_=32.2, p<0.0001), as well as morphology (Wing Morphology: F_4,204_=48.8, p<0.0001). Ellipses represent 90% confidence intervals. PC1 largely captures the extent to which a song is ancestral-like. Song characteristics of rattling are in many ways intermediate to those of ancestral and purring songs.


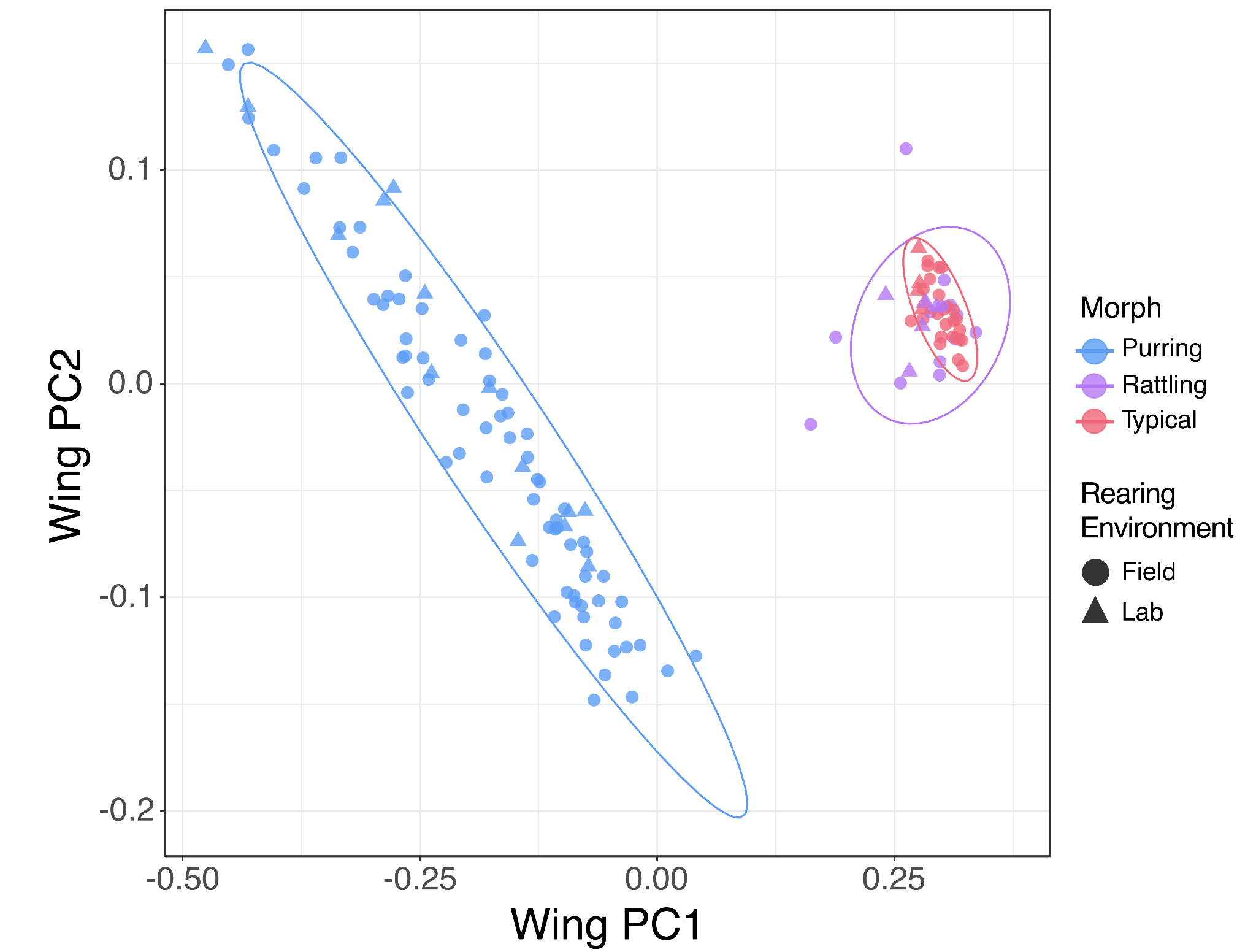


Figure S4. Morph-level differences in wing morphology (based on geometric morphometrics of 14 landmarks of the dorsal side of the wing; Figure S2, Table S2) were robust to differences in rearing environment. Morph was strongly predictive of morphological differences (MANOVA; Morph: F_4,248_=60.0, p<0.0001) while rearing treatment and the interaction between morph and rearing treatment were not (MANOVA: Rearing Treatment: F_2,123_=2.0, p=0.14; Morph x Rearing Treatment: F_4,248_=0.58, p=0.68). The consistency of morphological differences across morphs within a common-garden, lab context suggests that differences have a genetic basis.


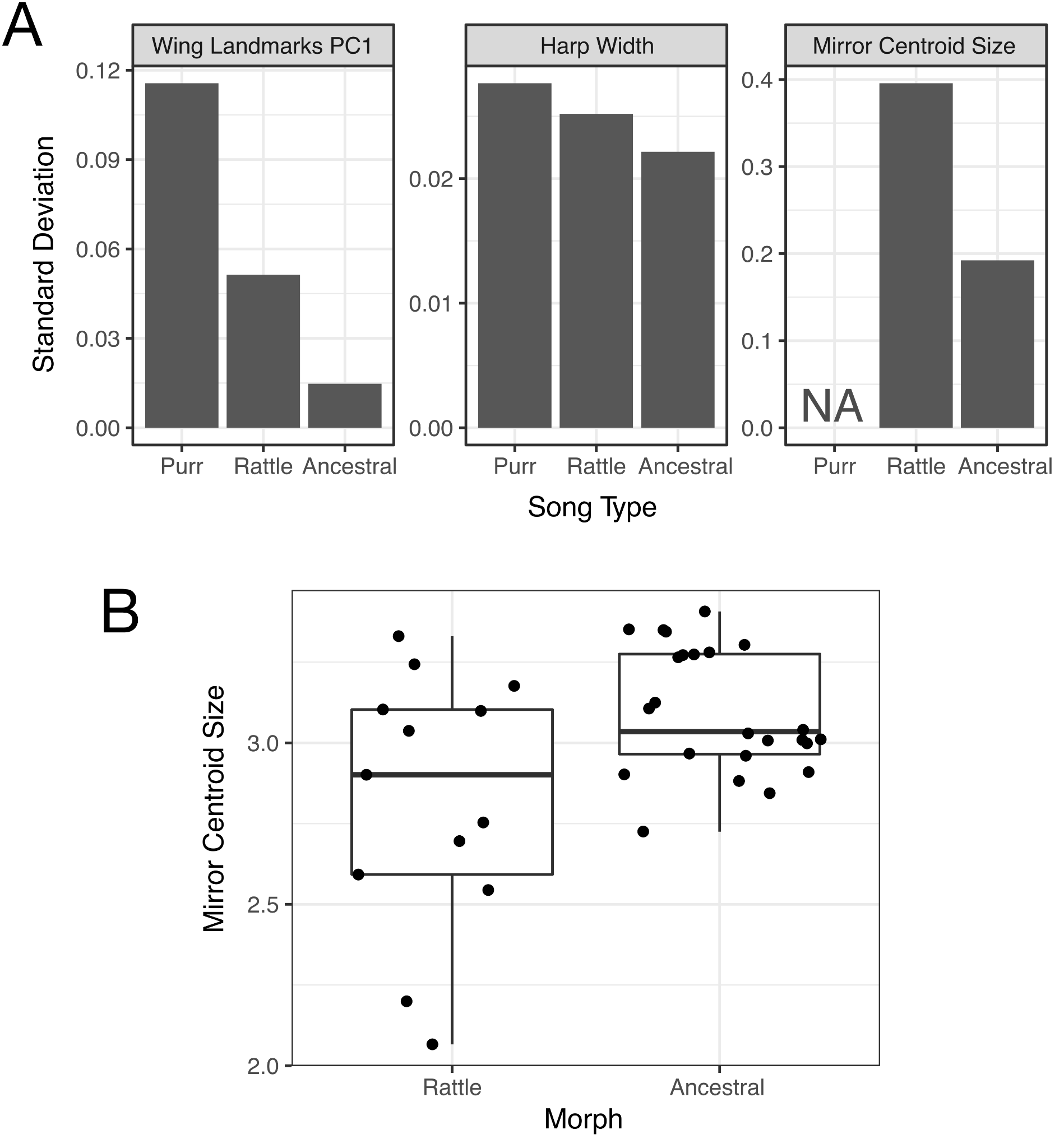


Figure S5: Differences in morphology among morphs. A) Standard deviations of wing morphometric variables show greater levels of morphological variation amongst alternate morphs (purring and rattling) compared to wings of ancestral males (Levine’s test: F_2,103_=21.2, p<0.0001). B) Though there is a statistically significant difference in mirror size between rattling and ancestral wings, it cannot explain the dramatic differences in song between morphs. There is much overlap in mirror size between morphs, and morph-level differences became non-significant when two outlier rattling males (with small mirrors) were removed from the dataset. Additionally, a larger resonator (mirror) should be associated with lower frequency sound [(Bennet-Clark 1999)](https://paperpile.com/c/7lU1tu/HQIbB), but we found the opposite pattern in rattling, but not ancestral, males—rattling songs have higher mean frequency than ancestral songs.


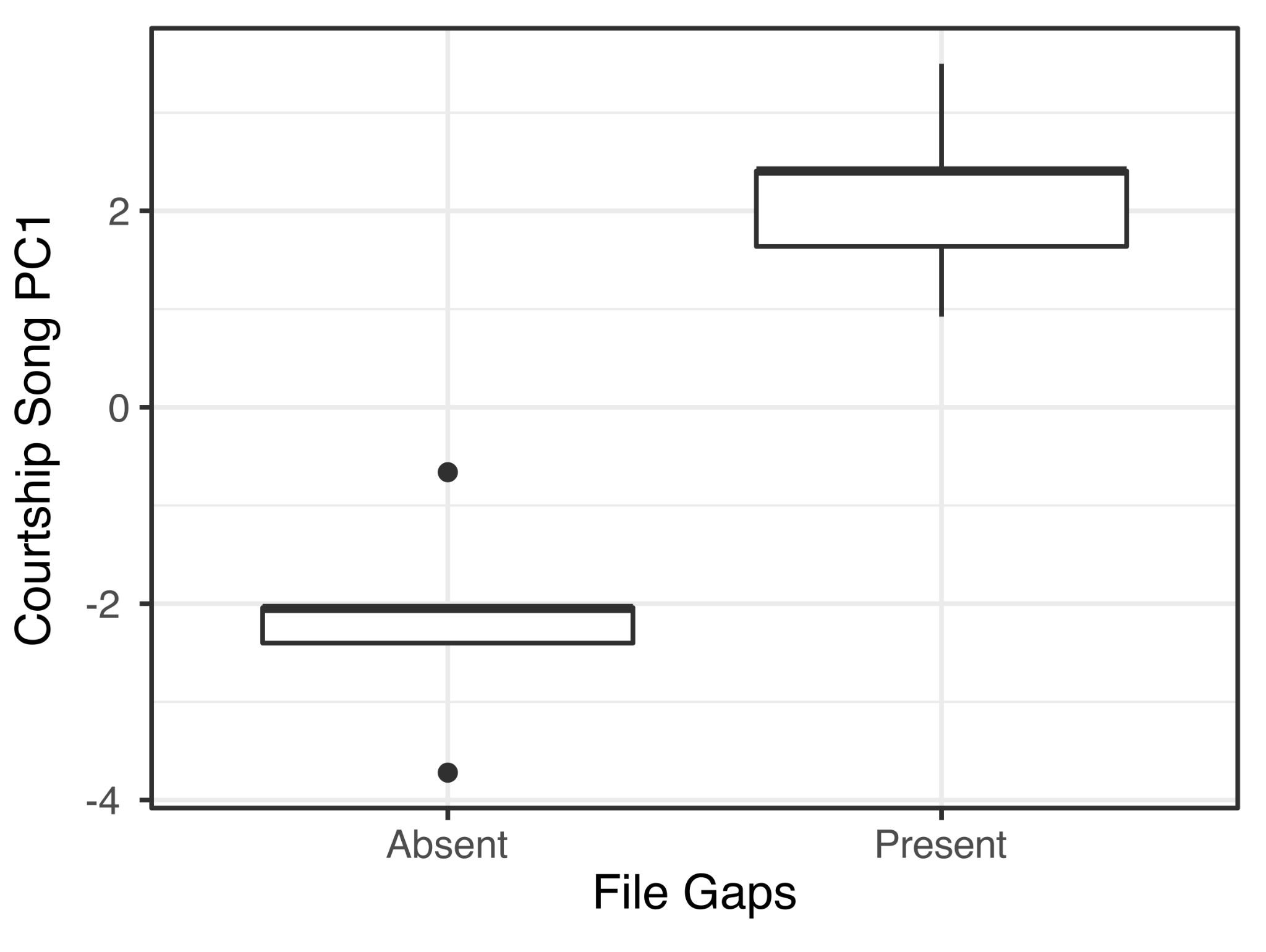


Figure S6. Courtship songs of second-generation, lab-reared males differed greatly depending on the presence of file tooth gaps, a diagnostic characteristic of the rattling morph. Males with tooth gaps (rattling males) had significantly greater courtship song PC1 values than males without gaps (ancestral males; T-test: t=6.68, df=7.88, p=0.0002, n=10). Courtship songs and wings were analyzed within two weeks of males eclosing to the adult stage in a common-garden, lab setting, removing most environmental/age effects on phenotypic differences.

Supplemental Tables

| Table S1. Sampling sites across Hawaii used in this study. | | | | |
| --- | --- | --- | --- | --- |
| **Site Name** | **Island** | **Males (N)** | **Females (N)** | **Location** |
| Hilo | Hawai’i | 35 | 30 | University of Hawai’i at Hilo |
| Kalaupapa | Moloka’i | 7 | 26 | Kalaupapa National Historical Park |
| Manoa | O’ahu | 31 | 30 | University of Hawai’i at Manoa, Astronomy Center |
| La’ie | O’ahu | 26 | 30 | Brigham Young University – Hawaii |
| Wailua | Kaua’i | 28 | 31 | Kaua’i Research Station at the University of Hawai’i College of Tropical Agriculture |
| Kapa’a | Kaua’i | 26 | 25 | Kapa’a Pono Kai Resort |

| Table S2. Descriptions and notes for wing landmarks. | | |
| --- | --- | --- |
| **Landmark #** | **Description** | **Notes** |
| 1 | Apical tip of Cu1 |  |
| 2 | Apical tip of scraper | If no scraper, directly below wing bend |
| 3 | Basal tip of scraper | If no scraper, on top of landmark 2 |
| 4 | Basal point of Cu2 where the vein begins to curve away from Cu1 | Usually proximal end of the file and basal point of the harp, where Cu1 and Cu2 are no longer parallel |
| 5 | Distal corner of the harp | In ancestral wings there is usually a junction of Cu2 and distal harp vein. In flatwings (wings with reduced harps and no mirrors), distal harp vein often appears to merge seamlessly with Cu2 |
| 6 | Basal junction of mirror and distal harp vein | Placed on top of landmark 11 if mirror is absent |
| 7 | Basal junction of upper and lower mirror cells | Placed on top of landmark 11 if mirror is absent |
| 8 | Distal junction of apical accessory vein and upper mirror cell | Placed on top of landmark 11 if mirror is absent |
| 9 | Proximal junction of apical accessory vein and upper mirror cell | Placed on top of landmark 11 if mirror is absent |
| 10 | Apical junction of upper and lower mirror cells | Placed on top of landmark 11 if mirror is absent |
| 11 | Junction of lateral vein and distal harp vein | Lateral vein is the most basal connector between Cu1 and distal harp vein. In ancestral, this is at the proximal edge of the mirror. In flatwings, the lateral vein may not be at the apical tip of the harp |
| 12 | Junction of lateral vein and Cu1 | Lateral vein is the most basal connector between Cu1 and distal harp vein. This will always be across from landmark 11 |
| 13 | Apical tip of extended harp | May overlap with landmark 12 in many wings, including most wings with a mirror |
| 14 | Basal, proximal tip of harp on Cu1 | Adjacent to landmark #4 |

| Table S3. Means and standard deviations of trait values of ‘ancestral’, ‘rattling’, and ‘purring ’individuals from the dataset used in Figure S3. | | | | |
| --- | --- | --- | --- | --- |
|  |  | Ancestral (n = 24) | Rattling (n = 13) | Purring (n = 69) |
| Calling Song (composite variables) |  |  |  |  |
|  | PC1 | -3.665 (1.101) | -0.216 (0.428) | 1.384 (0.404) |
|  | PC2 | -0.386 (0.534) | 1.292 (0.776) | -0.261 (0.784) |
| Calling Song (individual characteristics) |  |  |  |  |
|  | Amplitude | 126.434 (82.566) | 15.497 (6.263) | 1.539 (0.609) |
|  | Dominant Frequency | 4877.105 (191.528) | 5805.923 (1702.966) | 9204.915 (3942.025) |
|  | rangeA | 0.014 (0.014) | 0.030 (0.02) | 0.154 (0.041) |
|  | rangeB | 0.713 (0.095) | 0.300 (0.073) | 0.152 (0.034) |
|  | rangeC | 0.083 (0.032) | 0.282 (0.072) | 0.193 (0.039) |
|  | rangeD | 0.100 (0.053) | 0.137 (0.039) | 0.173 (0.037) |
|  | rangeE | 0.059 (0.027) | 0.160 (0.074) | 0.170 (0.032) |
|  | rangeF | 0.031 (0.017) | 0.091 (0.073) | 0.157 (0.037) |
|  | Freq. Evenness | -0.271 (0.043) | -0.121 (0.03) | -0.036 (0.024) |
| Courtship Song (composite variables) |  |  |  |  |
|  | PC1 | 4.841 (1.303) | -0.002 (0.771) | -2.448 (0.51) |
|  | PC2 | -0.055 (0.149) | -0.502 (0.586) | 0.058 (1.903) |
| Courtship song (individual characteristics) |  |  |  |  |
|  | Amplitude (chirp) | 172.234 (85.612) | 8.030 (5.037) | 2.850 (3.162) |
|  | Dominant Frequency (chirp) | 4868.318 (183.622) | 4859.500 (477.827) | 8051.233 (4186.844) |
|  | rangeA (chirp) | 0.006 (0.003) | 0.019 (0.016) | 0.129 (0.053) |
|  | rangeB (chirp) | 0.779 (0.084) | 0.357 (0.071) | 0.154 (0.041) |
|  | rangeC (chirp) | 0.073 (0.037) | 0.228 (0.053) | 0.183 (0.059) |
|  | rangeD (chirp) | 0.074 (0.037) | 0.114 (0.033) | 0.184 (0.044) |
|  | rangeE (chirp) | 0.041 (0.023) | 0.138 (0.036) | 0.189 (0.079) |
|  | rangeF(chirp) | 0.026 (0.015) | 0.144 (0.043) | 0.160 (0.053) |
|  | Freq. Evenness (chirp) | -0.302 (0.039) | -0.121 (0.028) | -0.055 (0.036) |
|  | Amplitude (trill) | 113.409 (52.582) | 7.971 (4.745) | 1.795 (0.952) |
|  | Dominant Frequency (trill) | 4772.864 (168.234) | 4909.200 (583.036) | 7343.548 (3906.386) |
|  | rangeA (trill) | 0.009 (0.005) | 0.026 (0.029) | 0.142 (0.044) |
|  | rangeB (trill) | 0.716 (0.098) | 0.348 (0.077) | 0.170 (0.03) |
|  | rangeC (trill) | 0.104 (0.052) | 0.242 (0.053) | 0.190 (0.051) |
|  | rangeD (trill) | 0.097 (0.053) | 0.120 (0.033) | 0.182 (0.04) |
|  | rangeE (trill) | 0.039 (0.018) | 0.138 (0.033) | 0.170 (0.037) |
|  | rangeF (trill) | 0.035 (0.017) | 0.127 (0.035) | 0.145 (0.035) |
|  | Freq. Evenness (trill) | -0.274 (0.041) | -0.118 (0.035) | -0.041 (0.024) |
| Wing Morphology |  |  |  |  |
|  | PC1 | 0.305 (0.015) | 0.283 (0.051) | -0.170 (0.116) |
|  | PC2 | 0.035 (0.014) | 0.028 (0.032) | -0.021 (0.078) |
|  | Scraper (proportion present) | 24/24 | 12/13 | 55/68 *scraper presence undetermined for one male due to obscured photo |
|  | Mirror (proportion present) | 24/24 | 13/13 | 0/69 |
|  | Harp Width | 0.271 (0.022) | 0.281 (0.025) | 0.138 (0.028) |
|  | Mirror Size | 3.098 (0.192) | 2.826 (0.396) | NA |

| Table S4: Correlations (r) between song and wing traits within A) ancestral (N = 23), B) rattling (N = 13), and C) purring males (N = 69) from the dataset used in Figure S3. P-values shown in parentheses. All variables are continuous except scraper (presence/absence). Bold cells highlight significant morphology-song relationships. Note that we show correlations for scraper presence in rattling males, but these patterns are driven by a single rattling male that lacked a scraper and should be interpreted with caution. | | | | |
| --- | --- | --- | --- | --- |
| *A) Ancestral males* | | | | |
|  | **Wing PC1** | **Scraper** | **Mirror Size** | **Harp Width** |
| **Calling Song PC1** | -0.177 (0.483) | NA | -0.038 (0.881) | 0.014 (0.957) |
| **Peak Frequency** | 0.198 (0.432) | NA | -0.157 (0.534) | 0.079 (0.757) |
| **Amplitude** | -0.107 (0.673) | NA | 0.121 (0.632) | 0.111 (0.660) |
| **Frequency Evenness** | -0.269 (0.280) | NA | 0.046 (0.858) | 0.047 (0.852) |
| *B) Rattling males* | | | | |
|  | **Wing PC1** | **Scraper** | **Mirror Size** | **Harp Width** |
| **Calling Song PC1** | -0.148 (0.630) | -0.159 (0.604) | -0.206 (0.500) | 0.227 (0.457) |
| **Peak Frequency** | -0.228 (0.453) | 0.195 (0.524) | -0.611 (**0.027**) | -0.294 (0.329) |
| **Amplitude** | -0.298 (0.323) | -0.448 (0.125) | -0.093 (0.764) | 0.107 (0.728) |
| **Frequency Evenness** | -0.169 (0.582) | -0.368 (0.217) | -0.151 (0.623) | 0.001 (0.998) |
| *C) Purring males* | | | | |
|  | **Wing PC1** | **Scraper** | **Mirror Size** | **Harp Width** |
| **Calling Song PC1** | -0.227 (0.084) | -0.417 (**0.001**) | NA | -0.084 (0.528) |
| **Peak Frequency** | -0.234 (0.074) | -0.513 (**<0.0001**) | NA | -0.144 (0.275) |
| **Amplitude** | 0.096 (0.465) | -0.170 (0.199) | NA | 0.234 (0.072) |
| **Frequency Evenness** | -0.159 (0.225) | 0.025 (0.851) | NA | -0.231 (0.076) |

| Table S5. Pairwise comparisons of the effects of song stimuli (purring, rattling, ancestral, and white noise (WN)) on A) female cricket phonotactic behavior and B) contact with playback speaker. Comparisons made with estimated marginal means, and contrasts from Firth’s Penalized Logistic Regression for phonotactic behavior and contact with speaker models, respectively (N = 30 females from Hilo) | | | | |
| --- | --- | --- | --- | --- |
| ***A) Phonotaxis*** | | | | |
| **Contrast** | **estimate** | **SE** | **z-ratio** | **P** |
| Purr:Rattle | -1.68 | 0.463 | -3.619 | **0.0017** |
| Purr:Typical | -3.77 | 0.579 | -6.512 | **<0.0001** |
| Purr:WN | 1.58 | 1.052 | 1.504 | 0.4353 |
| Rattle:Typical | -2.09 | 0.648 | -3.231 | **0.0068** |
| Rattle:WN | 3.26 | 1.119 | 2.912 | **0.0188** |
| Typical:WN | 5.35 | 1.177 | 4.547 | **<0.0001** |
| ***B) Contact with Speaker*** | | | | |
| **Contrast** | **estimate** | **SE** | **Chisq** | **P** |
| Purr:Rattle | 1.215 | 0.550 | 4.294 | **0.038** |
| Purr:Typical | 3.722 | 0.491 | Inf | **<0.0001** |
| Purr:WN | -1.362 | 1.475 | 1.352 | 0.245 |
| Rattle:Typical | 2.507 | 0.629 | 19.968 | **<0.0001** |
| Rattle:WN | -2.577 | 1.526 | 5.323 | **0.021** |
| Typical:WN | -5.084 | 1.506 | 40.237 | **<0.0001** |

Supplementary Methods

Sampling

To collect crickets, we used an unbiased sweeping method in the fields at each location, capturing crickets visually. This method does not use sound to locate crickets and so allows us to collect all sexes and morphs equally (i.e., louder males are not “hunted down”), and has been used for many years in previous work [(Tinghitella et al. 2018; Tinghitella et al. 2021)](https://paperpile.com/c/7lU1tu/oMWf+C1jr).

Song Analysis

For courtship songs we selected and measured characteristics of the chirp and trill separately, as their sound properties differ from one another [(Hoy 1974; Bennet-Clark 2003)](https://paperpile.com/c/7lU1tu/4HTgb+fwgaC), and it’s possible that selection on behavior could shape the two parts of the song in different ways. There is an incredible amount of variation in the length of a trill, and so analyzing the entire courtship song together may have heavily weighted results for one part over the other in our analyses. Before measuring song characteristics, we used Audacity to apply a high-pass filter at 1500 Hz (roll-off: 48 dB per octave) to remove background frequencies well below the hearing range of *T. oceanicus* [(Hoy et al. 1982)](https://paperpile.com/c/7lU1tu/8QNT8) and because we did not detect any song frequencies below this in our recordings. We measured the relative amplitude of six different frequency ranges in order to give us a much more detailed understanding of song frequency composition (as opposed to solely measuring dominant frequency), which was important because purring songs are quite broadband and frequency composition varies greatly among individual purring males [(Tinghitella et al. 2018)](https://paperpile.com/c/7lU1tu/oMWf). For each frequency range, we applied a bandpass filter with a steep slope to eliminate frequencies outside the desired range (MBandPass 13.01, MeldaProduction, settings: Q=1.00, slope=96), then measured the amplitude of the sound. For our measure of how broadband songs were (frequency evenness), a greater value indicates greater frequency evenness within the song, meaning that acoustic energy is distributed more evenly across frequency ranges, resulting in a more broadband sound.

In order to measure amplitude from calling and courtship song recordings, we also measured the overall amplitude (and the amplitude of each frequency range) of ambient noise in the recording room each night to use as a 0 dB reference. Any males with overall song amplitudes at or below this threshold (undetectable over ambient noise) were deemed non-sound-producing and excluded from further analyses. Decibels are measured on a logarithmic scale, so we converted our amplitude measurements in dB to their amplitude ratio (a linear format) prior to subsequent analyses [(Brown and Riede 2017)](https://paperpile.com/c/7lU1tu/gv4gb), as is standard in bioacoustics work (e.g., [Broder et al. 2021; Tinghitella et al. 2021)](https://paperpile.com/c/7lU1tu/C1jr+9v6Ly). Courtship song PCAs used characteristics from both the chirp and trill.

Wing Morphometrics Repeatability

After a training and quality checking period with an experienced landmarker, two independent observers placed landmarks on the photos of the right wing of each male using tpsDIG2. For any wings where the Procrustes distance between the two observers was > 0.3, the wing was rescored by an expert observer (N=18 wings) and these new measurements were instead used in the final dataset. The expert observer then rescored a random subset of 10 wings. We compared each landmark from these 10 wings among observers—the initial observer’s data whose measurements most closely matched those of the expert observer were used in the final dataset.

Morphology and Performance of Novel Morphs

When we examined wings using microscopy, we observed and needed to define a “gap.” To determine a threshold for what was considered a gap between teeth, we measured the distance between teeth on five ancestral male wings. All ancestral teeth were spaced < 0.02 mm apart, so we conservatively defined gaps as any spacing between teeth > 0.03 mm. We measured the total file length, number of gaps, and proportion of the file that was made up of gaps (sum of length of gaps divided by total file length).

For the phonotaxis experiments using female crickets and flies from Hilo, we played purring, ancestral, and rattling calling songs. Using a PCA based on the same nine calling song characteristics described above (Figure S2), we selected and used the rattling song with the most central (closest to the origin) sound characteristics for use in the phonotaxis experiments.

Common Garden Rearing

In July 2021, we collected eggs from field-caught individuals from Hilo, Manoa, and Kapa’a and reared them for two generations in the lab to reduce the impact of plastic or transgenerational effects [(Kawecki and Ebert 2004)](https://paperpile.com/c/7lU1tu/FWhiG), following Pascoal et al. ([2014)](https://paperpile.com/c/7lU1tu/opxk). To ensure that morph differences were not strictly due to rearing conditions, we reared crickets for two generations in common garden and found that the same morph-level clustering exists in lab-reared animals (Figure S4). To test the possibility that rattling morphology is environmentally-induced, we first compared the proportion of males expressing rattling morphology in the field (N=8/31 males; 26% rattling; identified using song and wing characteristics identified in clustering analysis, as well as file tooth gaps) to proportions from the first-generation lab reared males (N=13/48; 27% rattling). We next recorded courtship songs of second generation lab–born rattling males and used a t-test to compare their courtship song PC1 values to those of rattling males recorded in the field. Finally, to further compare field and lab-reared animals across all morphs, we measured wing morphometrics (using landmarking methods described above) from newly-eclosed, lab-born males (rattling: N=5; ancestral: N=5; purring: N=14; all source populations had been in the lab for at least two generations), and ran a MANOVA, with the first two axes of morphometric PCA as the response variables, and morph, rearing treatment (lab vs. field-born), and their interaction as predictor variables. A significant interaction term in this model would suggest that morph-level differences in wing morphology are dependent upon environmental (i.e., rearing) differences. In all instances, we recorded male courtship song and excised right wings from lab-reared males within two weeks of their eclosion to the adult stage to remove phenotypic differences due to natural, age-related wear.

References

[Bennet-Clark, H. C. 1999. Resonators in insect sound production. J. Exp. Biol. 202:3347–3357.](http://paperpile.com/b/7lU1tu/HQIbB)

[Bennet-Clark, H. C. 2003. Wing resonances in the Australian field cricket Teleogryllus oceanicus. J. Exp. Biol. 206:1479–1496.](http://paperpile.com/b/7lU1tu/fwgaC)

[Broder, E. D., A. W. Wikle, J. H. Gallagher, and R. M. Tinghitella. 2021. Substrate-borne vibration in Pacific field cricket courtship displays. JOR 30:43–50. Pensoft Publishers.](http://paperpile.com/b/7lU1tu/9v6Ly)

[Brown, C., and T. Riede. 2017. Comparative Bioacoustics: An Overview. Bentham Science Publishers.](http://paperpile.com/b/7lU1tu/gv4gb)

[Desutter-Grandcolas, L. 1998. Broad-frequency modulation in cricket (Orthoptera, Grylloidea) calling songs: two convergent cases and a functional hypothesis. Can. J. Zool. 76:2148–2163. NRC Research Press.](http://paperpile.com/b/7lU1tu/6bYqq)

[Duncan, J., C. D. Soulsbury, and F. Montealegre-Z. 2021. Differentiation between left and right wing stridulatory files in the field cricket Gryllus bimaculatus (Orthoptera: Gryllidae). Arthropod Struct. Dev. 65:101076.](http://paperpile.com/b/7lU1tu/PeYAZ)

[Hoy, R. R. 1974. Genetic Control of Acoustic Behavior in Crickets. Integr. Comp. Biol. 14:1067–1080. Oxford Academic.](http://paperpile.com/b/7lU1tu/4HTgb)

[Hoy, R. R., G. S. Pollack, and A. Moiseff. 1982. Species-Recognition in the Field Cricket, Teleogryllus oceanicus: Behavioral and Neural Mechanisms. Am. Zool. 22:597–607. academic.oup.com.](http://paperpile.com/b/7lU1tu/8QNT8)

[Imaizumi, K., and G. S. Pollack. 1999. Neural Coding of Sound Frequency by Cricket Auditory Receptors. J. Neurosci. 19:1508–1516.](http://paperpile.com/b/7lU1tu/KhMyV)

[Kawecki, T. J., and D. Ebert. 2004. Conceptual issues in local adaptation. Ecol. Lett. 7:1225–1241. Wiley.](http://paperpile.com/b/7lU1tu/FWhiG)

[Montealegre-Z, F., T. Jonsson, and D. Robert. 2011. Sound radiation and wing mechanics in stridulating field crickets (Orthoptera: Gryllidae). J. Exp. Biol. 214:2105–2117.](http://paperpile.com/b/7lU1tu/Y36g3)

[Montealegre-Z, F., J. F. C. Windmill, G. K. Morris, and D. Robert. 2009. Mechanical phase shifters for coherent acoustic radiation in the stridulating wings of crickets: the plectrum mechanism. J. Exp. Biol. 212:257–269.](http://paperpile.com/b/7lU1tu/OVOuV)

[Pascoal, S., T. Cezard, A. Eik-Nes, K. Gharbi, J. Majewska, E. Payne, M. G. Ritchie, M. Zuk, and N. W. Bailey. 2014. Rapid convergent evolution in wild crickets. Curr. Biol. 24:1369–1374.](http://paperpile.com/b/7lU1tu/opxk)

[Pascoal, S., M. Mendrok, A. J. Wilson, J. Hunt, and N. W. Bailey. 2017. Sexual selection and population divergence II. Divergence in different sexual traits and signal modalities in field crickets (Teleogryllus oceanicus). Evolution 71:1614–1626.](http://paperpile.com/b/7lU1tu/yN9fo)

[Tinghitella, R. M., E. D. Broder, J. H. Gallagher, A. W. Wikle, and D. M. Zonana. 2021. Responses of intended and unintended receivers to a novel sexual signal suggest clandestine communication. Nat. Commun. 12:797.](http://paperpile.com/b/7lU1tu/C1jr)

[Tinghitella, R. M., E. D. Broder, G. A. Gurule-Small, C. J. Hallagan, and J. D. Wilson. 2018. Purring Crickets: The Evolution of a Novel Sexual Signal. Am. Nat. 192:773–782.](http://paperpile.com/b/7lU1tu/oMWf)

[Zuk, M., J. T. Rotenberry, and R. M. Tinghitella. 2006. Silent night: Adaptive disappearance of a sexual signal in a parasitized population of field crickets. Biol. Lett. 2:521–524.](http://paperpile.com/b/7lU1tu/6YLRP)
